# Studies of Social Anxiety Using Ambulatory Assessment: Systematic Review

**DOI:** 10.2196/46593

**Published:** 2024-04-04

**Authors:** Javier Fernández-Álvarez, Desirée Colombo, Juan Martín Gómez Penedo, Maitena Pierantonelli, Rosa María Baños, Cristina Botella

**Affiliations:** 1 Department of Basic and Clinical Psychology and Psychobiology Jaume I University Castellon de la Plana Spain; 2 Fundación Aiglé Buenos Aires Argentina; 3 Facultad de Psicología Universidad de Buenos Aires (CONICET) Buenos Aires Argentina; 4 Polibienestar Research Institute University of Valencia Valencia Spain; 5 Department of Personality, Evaluation, and Psychological Treatments University of Valencia Valencia Spain; 6 Ciber Fisiopatologia Obesidad y Nutricion (CB06/03 Instituto Salud Carlos III) Madrid Spain

**Keywords:** social anxiety disorder, ambulatory assessment, ecological momentary assessment, intensive longitudinal methods, systematic review, social anxiety, use, qualitative synthesis, emotional, cognitive, behavioral, development, mental disorder, anxiety, mental health, mobile health, mHealth, monitoring, review, assessment, mobile phone

## Abstract

**Background:**

There has been an increased interest in understanding social anxiety (SA) and SA disorder (SAD) antecedents and consequences as they occur in real time, resulting in a proliferation of studies using ambulatory assessment (AA). Despite the exponential growth of research in this area, these studies have not been synthesized yet.

**Objective:**

This review aimed to identify and describe the latest advances in the understanding of SA and SAD through the use of AA.

**Methods:**

Following the PRISMA (Preferred Reporting Items for Systematic Reviews and Meta-Analyses) guidelines, a systematic literature search was conducted in Scopus, PubMed, and Web of Science.

**Results:**

A total of 70 articles met the inclusion criteria. The qualitative synthesis of these studies showed that AA permitted the exploration of the emotional, cognitive, and behavioral dynamics associated with the experience of SA and SAD. In line with the available models of SA and SAD, emotion regulation, perseverative cognition, cognitive factors, substance use, and interactional patterns were the principal topics of the included studies. In addition, the incorporation of AA to study psychological interventions, multimodal assessment using sensors and biosensors, and transcultural differences were some of the identified emerging topics.

**Conclusions:**

AA constitutes a very powerful methodology to grasp SA from a complementary perspective to laboratory experiments and usual self-report measures, shedding light on the cognitive, emotional, and behavioral antecedents and consequences of SA and the development and maintenance of SAD as a mental disorder.

## Introduction

### Background

Social anxiety (SA) is a normal and adaptive manifestation that all human beings experience in anticipation of a potential interactional threat. Similar to any adaptive response, it is enormously beneficial, particularly in protecting people from potential dangers that may arise from social interactions [1]. However, this adaptive response is sometimes exacerbated, and instead of preparing the individual for optimal performance, it becomes paralyzing, triggering intense fear, catastrophic thoughts, and avoidance behaviors, among other characteristic manifestations. When this pathological response becomes habitual in anticipation and confrontation of social interactions, it is referred to as SA disorder (SAD).

Accordingly, SAD is understood as a prevalent clinical condition characterized by intense fear and avoidance of social situations. SAD is a heterogeneous clinical condition that usually entails high levels of dysfunction in the lives of people who experience it. This heterogeneous nature of SAD is marked by the dynamic deployment of cognitions, emotions, and behaviors in the interaction with others and in different contexts [2].

### Ambulatory Assessment

Ambulatory assessment (AA) serves as an umbrella term that includes specific techniques such as experience sampling methods, ecological momentary assessment, or daily retrospective methods. These techniques constitute a research methodology of paramount significance in the field of clinical psychology and psychotherapy, enabling researchers and clinicians to gather in-depth, ecologically valid data from individuals. Unlike traditional assessment methods that rely on retrospective self-reporting, AA involves the repeated real-time measurement and gauging of various aspects of an individual’s experiences, behaviors, and physiological responses. The fact that it entails multiple assessments over a certain period makes AA an optimal tool to explore within-person fluctuations and trajectories of these experiences and behaviors.

Moreover, AA circumvents the biases that usual retrospective reports may have because it is usually implemented in momentary assessments or recent retrospective reports (eg, daily diaries [3]). Owing to the variability of psychological processes such as affective and emotional dynamics [4], AA can more precisely determine the fluctuation of symptoms. In addition, owing to the possibility of incorporating sensors and biosensors, AA can provide multimodal assessment, overcoming the biases of self-reports [5].

Over the last few years, there has been an increasing interest in exploring the use of AA in clinical psychology and psychotherapy. Owing to the incorporation of digital technologies, namely, mobile phones, AA has become a very powerful add-on for clinical researchers [6], first and foremost due to the possibility that AA provides of capturing data in real time, thus grasping contextual aspects from naturalistic settings that laboratory-based assessment does not allow for [7,8]. SA symptoms are particularly contextually bound, and thus, it is of utmost relevance to consider the sensitivity of the context [2].

All these characteristics have a central and common pursuit to personalize models of psychopathology [9]. There is no doubt that every person has a certain and unique composition of traits that may lead to functional and dysfunctional states in continuous interaction with the context. Therefore, the revolution of AA is helping foster the creation of tailored models with intensive longitudinal data, which can shed light on the factors that may lead people to experience and behave in adaptive or maladaptive ways.

In this sense, AA proves to be a suitable tool for capturing the co-occurrence of symptoms and psychological processes, offering crucial insights into the antecedents and consequences of SA with enhanced accuracy. This, in turn, facilitates a comprehensive understanding of the factors contributing to the appearance and maintenance of SA and SAD. Given the dimensional nature of SA, AA can shed light on the differences among clinical, subclinical, and healthy participants.

The understanding of SAD in ecological settings may also lead to improving psychological treatments. The insights gained through AA can contribute to refining therapeutic approaches by investigating mechanisms of change [10]. However, to the best of our knowledge, no systematic review has synthesized the available evidence analyzing the strengths and limitations of the current state of the art.

### This Study

For all the aforementioned reasons, AA provides a range of advantages that justify its exponential growth as a research methodology in clinical psychology. Although a plethora of research has implemented AA in individuals with SA, these data have not been synthesized yet. Hence, the main aim of this review was to identify studies that used AA to explore SA.

## Methods

This review followed the recommendations of the PRISMA (Preferred Reporting Items for Systematic Reviews and Meta-Analyses) statement [11]. The full protocol was registered before the data analysis [12].

### Literature Search

Scopus, PubMed, and Web of Science literature searches were conducted. The search strategy for PubMed is available in Multimedia Appendix 1 and was adapted to the syntax requirements of each database. No filters were included in the searches. The reference lists of eligible articles were manually reviewed to identify additional relevant publications.

### Inclusion Criteria

To be included, the studies had to fulfill the following criteria: (1) studies that focused on participants with SAD or SA symptoms; (2) studies that used AA to explore the dynamics of SA, correlates of SA, or the feasibility of AA for SA assessment; (3) articles that included any sort of discussion regarding SA in AA (this criterion was included given that some articles fulfilled all the previous criteria but featured no specific discussion on SA); (4) articles published in peer-reviewed journals in English; and (5) studies that presented at least one active AA or passive data collected via sensor signals (eg, geo-mapping, accelerometer, or GPS) from a phone or smartwatch multiple times per day. Articles were excluded if they (1) were not empirical studies; (2) used a child or adolescent sample; and (3) did not use a daily diary, experience sampling, or AA approach that included assessment and discussion of SA.

### Study Selection and Data Extraction

**Figure 1:**
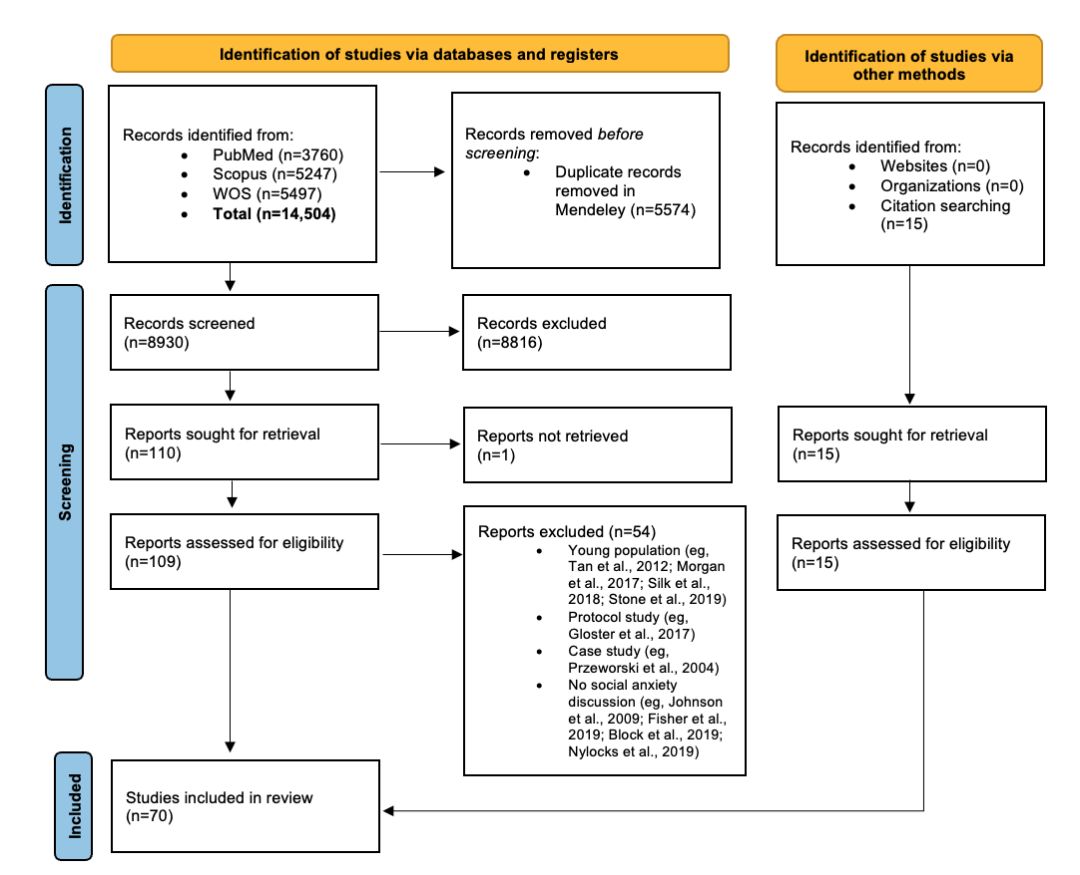
Systematic review search flow diagram. WOS: Web of Science.

A database search was conducted until August 31, 2023. The literature search produced a total of 14,504 articles, 8926 (61.54%) of which were retained after removing duplicates. A total of 1.23% (110/8926) of these articles were retrieved after title and abstract screening. The subsequent application of the selection criteria resulted in the inclusion of 50% (55/110) of the articles, adding another 15 studies that were identified through citation searching. In total, we included 70 studies (Figure 1). Studies were independently selected by 2 researchers, and disagreements were resolved through consensus.

Using premade tables, the following information was extracted: author and year of publication, study design, follow-up period and sample size and characteristics, aims of the study, measures collected through AA, app name in the case of smartphone-based AA, participation rate (ie, response to recruitment), attrition rate, compliance with AA questions, incentives, outcomes explored, AA measures, frequency of AA, and main findings.

Finally, to summarize the main information, 2 of the authors proposed categories. To form a category, it was required that at least 2 of the included studies addressed the same or a similar topic. To reach an agreement, the resulting categories underwent thorough discussion between the first and fourth authors. This criterion was applied to the “Principal themes explored” section. For the “Emerging topics” section, the criterion for category inclusion was based on relevance regarding addressing a key aspect of the literature that had not been explored before.

## Results

### Sample Characteristics

Of the 70 studies selected for this systematic review, 29 (41%) used the same sample (Textbox 1). Given that a large proportion of the studies were conducted in university settings, most of the included participants were undergraduate students and, therefore, young adults. Moreover, all the studies (70/70, 100%) were conducted in high-income countries, principally in the United States. A total of 43% (30/70) of the studies included both healthy and clinical populations. In total, 21% (15/70) of the studies were conducted only with clinical participants, whereas 36% (25/70) were conducted with healthy individuals exploring the dynamics of SA. In addition, 33% (23/70) of the studies included comorbid samples, mainly those with other anxiety or depressive disorders. Regarding gender, most studies (57/70, 81%) included more women than men. This reflects the prevalence rates of SAD, which have been shown to be higher in women than in men [13]. Table S1 in Multimedia Appendix 2 [14-81] and Table S2 in Multimedia Appendix 3 [14-81] summarize the characteristics and main findings of the included studies.

**Studies with the same samples.**

**Studies with the same samples**

- Blalock et al [14,15], Farmer and Kashdan [16], Goodman et al [17,18], Kashdan and Farmer [19], and Kashdan et al [20,21]
- Daniel et al [22,82,83] and Beltzer et al [23]
- Daniel et al [24], Daros et al [25], and Ladis et al [26]
- Doorley et al [27,28]
- Goodman et al [29-32]
- Oren-Yagoda et al [33-36]
- O’Toole et al [37,38]
- Villanueva et al [39] and Rinner et al [40]

### Methodological Characteristics of the Studies

#### Sampling Frequency

There were 2 types of sampling identified among the included studies. On the one hand, there were some studies that implemented a daily diary, which typically entails an end-of-day report. On the other hand, other AA studies comprised different numbers of daily assessments and diverse types of prompt contingencies. The duration of the AA ranged from 4 to 35 days. The most commonly implemented study duration was 14 days.

Although a large proportion of the studies (63/70, 90%) used a fixed or random scheduled prompt structure, a range of studies incorporated an event-contingent design [40-45]. Indeed, SA determinants, correlates, and consequences are particularly triggered in interpersonal situations, and event-contingent designs may be suitable for detecting relevant moments. Finally, studies such as those by Daniel et al [24], Helbig-Lang et [46], and Kashdan et al [20] included a mix of different types of data collection, combining random prompt, event-contingent, and end-of-day records.

#### Compliance

Compliance rates typically indicate the percentage of prompts or days that the participants completed on average. Compliance rates ranged from 40% to 95%, with average compliance across studies of 73.72% of prompts (SD 15.93%; median 80%).

#### Statistical Analysis

The vast majority of the studies (61/70, 87%) used hierarchical linear models to account for the nested nature of the collected data. A total of 4% (3/70) of the studies used a combination of hierarchical linear models and structural equation modeling [43,45,79], 1% (1/70) of the studies used machine learning techniques [47], 3% (2/70) of the studies calculated ANOVA models [23,42], and 1% (1/70) of the studies ran ordinary least squares regression [48].

### Methodological Design Characteristics

Table 1 summarizes the principal methodological characteristics of each study. Power analysis to calculate the needed sample size (which was rarely conducted; 15/70, 21% of the studies) and the psychometric properties of the AA questions (37/70, 53% of the studies) were the 2 criteria that were reported the least, whereas the percentage of AA compliance (56/70, 80% of the studies) and attrition rates of AA (46/70, 66% of the studies) were the criteria that were reported the most among the studies in this review.

**Table 1:** Methodological design characteristics.

Study	Compensation	Power analysis for sample size	Attrition rates	Psychometric properties of AA^a^	Compliance with AA
Arch et al [49]	Yes	+^b^	+	−^c^	+
Badra et al [50]	Yes	+	+	+	+
Bailey et al [51]	Yes	+	Unclear	−	+
Battista et al [52]	Yes	−	+	+	+
Beltzer et al [23]	Yes	−	−	+	−
Blalock et al [14]	Yes	−	+	+	+
Blalock et al [15]	No	−	+	−	+
Boukhechba et al [53]	Yes	−	−	−	−
Brown et al [54]	No	−	−	−	+
Brown et al [55]	Yes	−	+	−	−
Buckner et al [56]	No	−	+	+	+
Buckner et al [57]	Yes	−	+	−	+
Chow et al [58]	Yes	−	+	−	+
Čolić et al [59]	No	+	+	−	Unclear
Daniel et al [24]	No	−	+	−	+
Daniel et al [83]	Yes	−	+	−	Unclear
Daniel et al [82]	Yes	−	+	+	−
Daniel et al [22]	No	−	−	+	+
Daros et al [25]	Yes	−	+	−	+
Di Matteo et al [60]	Yes	−	−	+	−
Doorley et al [28]	Yes	−	+	+	Unclear
Doorley et al [27]	Yes	−	+	+	+
Farmer and Kashdan [61]	Yes	−	+	+	+
Farmer and Kashdan [16]	Yes	−	−	+	−
Geyer et al [62]	Yes	−	−	−	+
Goodman et al [63]	Yes	−	+	+	+
Goodman et al [17]	Yes	−	+	+	+
Goodman et al [18]	Yes	+	+	+	+
Goodman et al [32]	Yes	+	−	−	−
Goodman et al [30]	Yes	+	−	−	−
Goodman et al [31]	Yes	−	−	+	−
Goodman et al [29]	Yes	−	+	+	+
Goodman et al [64]	Yes	+	+	−	+
Hannah Lee [65]	Yes	−	+	−	+
Helbig-Lang et al [46]	No	−	−	+	−
Hur et al [66]	Yes	+	+	+	+
Jacobson et al [47]	No	+	+	+	N/A^d^
Jacobson and Bhattacharya [67]	No	−	−	+	−
Kane and Ashbaugh [68]	No	+	−	+	−
Kashdan and Steger [69]	No	−	+	+	−
Kashdan and Collins [70]	Yes	−	−	−	+
Kashdan et al [71]	No	−	+	−	+
Kashdan et al [20]	Yes	−	+	+	+
Kashdan et al [21]	Yes	−	+	+	+
Kashdan and Farmer [19]	Yes	−	+	−	+
Katz et al [41]	No	−	−	−	−
Kivity and Huppert [72]	Yes	−	+	−	+
Kim and Kwon [73]	Yes	+	+	−	+
Ladis et al [26]	Yes	−	+	−	+
Lee et al [42]	Yes	−	+	+	+
Nanamori et al [74]	Yes	−	−	+	+
Naragon-Gainey [43]	Yes	−	+	+	+
O’Grady et al [75]	Yes	−	+	−	+
Oren-Yagoda et al [35]	Yes	−	−	+	+
Oren-Yagoda and Aderka [33]	Yes	−	−	+	+
Oren-Yagoda et al [36]	Yes	−	−	+	+
Oren-Yagoda et al [34]	Yes	−	−	+	+
O’Toole et al [37]	Yes	−	+	+	+
O’Toole et al [38]	Yes	−	+	+	+
Papp et al [76]	Yes	−	−	−	+
Park and Naragon-Gainey [45]	Yes	−	+	−	+
Piccirillo and Robedaugh [77]	Yes	−	−	+	−
Reichenberger et al [78]	Yes	−	+	−	+
Rinner et al [40]	No	+	+	−	Unclear
Russell et al [44]	Yes	−	+	−	+
Saulnier et al [79]	Yes	−	−	+	+
Seah et al [48]	Yes	−	+	+	+
Villanueva et al [39]	No	+	+	−	Unclear
Walukevich-Dienst et al [80]	Yes	−	−	−	+
Wilson et al [81]	Yes	−	+	−	+

^a^AA: ambulatory assessment.

^b^Presence.

^c^Absence.

^d^N/A: not applicable.

### Principal Themes Explored

#### Affective and Emotional Dynamics

Naragon-Gainey [43] explored structural models of affect and internalizing symptoms. While the between-person variance in negative affect (NA) and concurrent levels of NA predicted SA, positive affect (PA) did not. This lack of significance contradicted many other AA studies that yielded significant between-person associations between lower levels of PA and higher levels of NA with SA [16,20,30,54,57,58,61,69,70].

Individuals with SAD showed not only higher overall levels of NA but also more emotional instability, which was not the case for PA [16]. These authors even suggested that the interaction between NA and instability could explain the appearance of SAD.

#### Discrete Emotions

As suggested by Rozen and Aderka [84], there is a wide range of discrete emotions that have not been integrated into classic models of SAD. In that sense, AA allows for a nuanced study of single emotions and how they are interconnected not only with each other but also with other cognitive-affective processes and behaviors.

First, Kashdan and Collins [70] revealed that SA was related to less time spent feeling happy and relaxed and more time spent feeling angry. The results also showed that happy moments were aroused in companion to others. The fact that SA was associated with fewer and less intense positive emotions and more anger episodes was independent of being with others or alone.

Oren-Yagoda et al [35] investigated the role of envy, showing that visual modes of communication are related to elevated envy compared to voice or text communication. In addition, envy predicted subsequent anxiety above and beyond previous anxiety as well as other negative emotions.

Loneliness is a defining emotion of people with SAD, and Oren-Yagoda et al [36] found that this relationship was also confirmed in an ecological setting using AA. A significant association was predicted in certain social situations (ie, negativity, positivity, and meaningfulness). Moreover, the relationship between loneliness and anxiety was shown to be reciprocal in individuals with SAD (loneliness predicted anxiety and anxiety predicted loneliness). This deleterious reciprocity was not found in healthy controls.

Finally, Oren-Yagoda et al [34] investigated the fluctuations of pride in individuals diagnosed with SAD. The results indicated that levels of pride were lower in patients with SAD than in nondiagnosed controls, although when pride was experienced, it predicted a reduction in anxiety levels.

#### Emotion Regulation

Emotion regulation (ER) was by far the most explored construct among the studies that implemented AA in individuals with SA. This may be explained by the fact that ER is a dynamic process, and AA is very suitable for detecting affect changes and fluctuations in daily life. In addition, not only are ER and SA symptoms highly context sensitive, but mounting research has also shown their interdependence [85], which justifies the importance of exploring how individuals with SAD use ER in daily life.

#### Discrete Strategies

Several studies explored only 1 strategy. For example, Kashdan et al [20] found that individuals with SAD experienced greater experiential avoidance than healthy individuals. Using the same sample, Kashdan et al [21] found that momentary experiential avoidance presented a stronger association with anxiety during social interactions for individuals with SAD than for individuals without SAD.

Similarly, another study explored the role of emotional suppression, demonstrating that, on days in which SA symptoms increased, the use of suppression tended also to increase [61,69]. The same was found by Beltzer et al [23], who identified that days with high levels of SA and expressive suppression led to fewer positive emotions.

Farmer and Kashdan [61] implemented a 2-week diary, showing that high SA was related to positive emotion suppression, fewer positive social events, and fewer positive emotions on subsequent days. In contrast, low SA was associated with fewer negative social events on the days after cognitive reappraisal was used to reduce distress. However, the use of cognitive reappraisal did not lead to any changes in people with high SA.

Meanwhile, Farmer and Kashdan [16] found that individuals with SAD were 3 times more likely to present acute shifts in NA, which may be a particular experience of this anxiety disorder and not others. This acute shift in NA may lead to experiencing emotions as uncontrollable and threatening, which in turn could explain the higher use of suppression.

Goodman et al [63] showed that alcohol consumption moderated the negative association between SA and a range of healthy social interactions such as laughter or feelings of acceptance; that is, SA was not related to the perceived quality of interpersonal interactions when participants consumed alcohol, suggesting that alcohol consumption may be a reinforcer of SA. These findings suggest that ER plays a central role in the experience of individuals with SAD who try to explicitly control their emotions and are aware of the effort they make to do so. In a different but related study, Goodman and Kashdan [29] found that both anxiety and pain were interfering factors in goal attainment, as well as finding an inverse association between daily meaning in life and perceived emotion-related goal interference.

#### Substance Use

Alcohol consumption and SAD are highly comorbid given that, among individuals with SAD, it is frequent to resort to alcohol as a coping strategy or, in other words, as an ER strategy. By means of AA, it is possible to detect how these 2 phenomena are interrelated. Therefore, several studies explored this topic. Battista et al [52] examined the relationship between alcohol consumption and SA, revealing that, after each alcoholic drink consumed, SA tended to decrease 2 hours later. Contrary to what the authors expected, this association was not explained by the level of trait SA.

Goodman et al [63] showed that alcohol consumption moderated the negative association between SA and a range of healthy social interactions such as laughter or feelings of acceptance; that is, alcohol consumption SA was no longer related to the perceived quality of interpersonal interactions, suggesting that SA may be a reinforcer of SA. This reinforcement could be either negative (attenuation of anxiety) or positive (better perception of social situations), which was the main finding of Goodman et al [64]. The results of this study yielded evidence supporting the negative reinforcement hypothesis such that people with SAD presented higher coping motives (negative reinforcers) but equal levels of affiliation motives (positive reinforcers).

Walukevich-Dienst et al [80] revealed that consuming substances as a coping strategy in SA is related to heavier consumption, especially on drinking days, which may be a risk factor for the development of an alcohol use disorder. Moreover, when there were SA coping motives, the consequences were more negative compared to days without SA coping motives. Interestingly, levels of SA at baseline were not moderators of these associations, indicating that the coping motive is more important than the antecedent levels of SA.

Kim and Kwon [73] showed that individuals with SAD had a higher increase rate of alcohol craving when they were tense and lonely and experiencing SA in comparison to individuals without a diagnosis of SAD. These results were moderated by the rate of rumination in the SAD group and avoidance in the non-SAD group. O’Grady et al [75] explored the role of social-contextual events in the relationship between trait SA and drinking. The results revealed a positive association between trait SA and drinking on the evenings of days in which the individuals experienced an embarrassing situation. This was significantly higher than in healthy participants, suggesting a behavioral maladaptive ER strategy to cope with the intensified levels of SA.

In addition, Buckner et al [56] studied the association between SA, cannabis use, cannabis craving, and situational variables. This study showed that SA interacted with cannabis craving predicting cannabis use, which sets out a complex relationship between SA and cannabis use and not a simple association (ie, cannabis use as a response to increased state SA).

In the study by Buckner et al [57], they showed that baseline SA was associated with increases in NA throughout the days in which the participants were monitored but was also significantly associated with postquit withdrawal. Participants with higher levels of SA presented more severe postquit withdrawal symptoms as well as an increase in NA during a cessation attempt. For this reason, the authors suggested that those participants may particularly benefit from intervention and treatment strategies.

Finally, Papp et al [76] found that students with higher levels of SA presented a stronger association between NA and prescription misuse. This study included externalizing and internalizing symptoms (depression and SA symptoms), and moderation was shown to be significant only for internalizing symptoms. This may indicate differential patterns of substance use as an ER strategy according to different personality traits.

#### The Complexity of ER: Polyregulation and Flexibility

Some studies incorporated a range of ER strategies in the AA process, which is in line with the current idea of *polyregulation*, that is, that individuals usually implement a range of strategies simultaneously [86]. For example, Daros et al [25] measured various strategies, and after clustering through a factorial analysis, 2 categories of ER strategies were considered for the analyses: avoidant and engagement strategies. However, the authors did not find any significant results for these 2 macrocategories.

On the basis of the model by Gross, Blalock et al [14] examined both suppression and cognitive reappraisal in SA in comparison to healthy individuals. Both groups presented worse emotional experiences when they suppressed positive versus negative emotions as well as when reappraising negative versus positive emotions. This suggests that suppression may be an adaptive strategy not to feel negative emotions, whereas reappraisal may be effective in increasing positive states. Interestingly, individuals with SAD showed more positive emotions after reappraising negative emotional states to feel fewer negative emotions than the healthy controls.

However, none of these previous examples explicitly framed their studies under the concept of emotion polyregulation, as did Ladis et al [26]. By including 8 strategies (problem-solving, introspection, distraction, acceptance, thought suppression, seeking advice, cognitive reappraisal, and expressive suppression), the authors systematically explored how often polyregulation occurs in daily life. Overall, the results yielded nonsignificant SA correlates of polyregulation, suggesting that it may be more dependent on within-person differences.

In addition to polyregulation, flexibility has been shown to be central in ER literature to distinguish adaptive from maladaptive regulatory processes [87]. Given that flexibility is mostly dependent on context, AA emerges as a very useful tool. O’Toole et al [38] investigated this specific topic in individuals with high and low SA and considered type and intensity as 2 contextual factors. This study revealed that SA moderated the relationship between emotion intensity and experiential avoidance. In individuals with high SA, there was a stronger association between experiential avoidance and specific emotions, such as guilt, nervousness, and sadness.

The study conducted by Beltzer et al [23] presents an illustrative example of how ER can be better explained by contextual triggers than by the adaptative or maladaptive continuum. The authors created an algorithm based on the 10 most used strategies to generate an ecological momentary intervention (EMI) based on contextual triggers. This algorithm was tested with a group of strategies (disengagement, engagement, and aversive cognitive perseveration) and with 10 individual single strategies compared with a random policy and a behavior policy, with ER effectiveness being the observed outcome. The contextual algorithm improved other policies in cases in which the top 10 strategies were considered separately. However, when the strategies were grouped into categories, the algorithm did not outperform the random recommender or the observed ER strategies.

Goodman et al [32] also explored ER flexibility considering 2 components of flexibility: the evaluation of contextual demands and matching regulatory strategies to contextual demands. The results indicated that people with SAD considered momentary assessments to be more anxiety provoking while presenting similar patterns to those of control participants when gauging contextual demands, particularly those related to perceived controllability. That is, some disengagement strategies (rumination, thought suppression, and expressive suppression) were found to be unrelated to perceived controllability. This means that these strategies were used independently of perceived controllability in a certain context. However, contrary to previous results, participants with SAD yielded similar patterns to those of control participants in response to anxiety intensity.

Higher anxiety ratings predicted greater use of all strategies regardless of the type of strategy used. In addition, this study showed that people with SAD may be more prone to use thought suppression but not engagement strategies, which is inconsistent with previous studies.

In other studies, a specific component of the heterogeneous and complex process of ER was explored. Although most clinical research on ER tends to simplify the discussion on putatively maladaptive (eg, expressive suppression) or adaptive (eg, cognitive reappraisal) strategies, there is a range of potential explanatory variables that set a more complex scenario than implementing certain strategies or not. For example, one study [24] investigated the perception of ER effectiveness, which is based on a robust research line that revolves around how goals shape and determine ER deployment and outcomes [88]. Daniel et al [24] found that, depending on the way in which effectiveness is measured (either judgment of effectiveness or change in affect), the results differ. While the judgment of effectiveness indicates that avoidance‐oriented ER attempts are less effective than engagement‐oriented ER attempts, changes in self-reported effects following ER attempts present the opposite results.

Similarly, Goodman et al [18] explored the extent to which beliefs of ER determine the use of specific patterns. In laboratory settings, De Castella et al [89] demonstrated that individuals with SAD presented low emotional self-efficacy, or the belief that emotions cannot be changed, but the results obtained by Goodman et al [18] provide ecological validity to a result that confirms the interdependency between cognitions and emotions [90].

Another approach is to calculate ER diversity, defined as the variety, frequency, and evenness of the implemented ER. Finally, Daniel et al [22] studied whether ER diversity predicted SA severity. The results showed that diversity within avoidance-oriented strategies was associated with both trait and state SA levels. At a more specific level of analysis, participants who responded more evenly and deployed a vast array of avoidance-oriented strategies more frequently were more prone to belonging to the high-SA group.

#### Emotion Clarity and Differentiation

Another important aspect that has been of increasing interest in the ER literature is linked to convergent processes such as emotion clarity and differentiation. Emotion clarity is defined as the ability to identify, distinguish, and describe specific emotions [91]. Park and Naragon-Gainey [45] found that lower momentary clarity was related to increases in subsequent momentary internalizing symptoms (ie, anxiety and depressive symptoms). This association was explained by an unsuccessful use of ER.

A total of 4% (3/70) of the studies examined emotion differentiation in individuals with SAD [19,37,48]. Emotion differentiation is conceptualized as the ability to recognize, identify, and label broad emotional experiences into discrete emotion categories [92]. Kashdan and Farmer [19] demonstrated that an increase in SA symptoms is linked to an impairment in negative emotion differentiation (ie, the ability to label and describe differences among negative emotions). In particular, it was found that negative emotion differentiation plays a relevant role in implementing more effective ER strategies. For example, Seah et al [48] conducted 2 studies that showed that negative emotion differentiation moderated the positive relationship between rumination and social avoidance. Similarly, O’Toole et al [37] showed that individuals with both high SA and poor negative emotion differentiation presented the least use of cognitive reappraisal. In addition, individuals with high SA used more suppression strategies despite the ability to differentiate positive emotions.

#### Perseverative Cognition and Mind Wandering

Although the most common variable that has been studied in individuals with SAD is postevent processing (PEP), there is a great overlap between PEP and rumination. In essence, both revolve around the perseverative thinking of past events, but PEP is strictly related to social interactions, including an important component of the actual interventions that both the individual and the others may have done or said. In total, 6% (4/70) of the studies used AA to examine processes related to PEP and rumination.

Helbig-Lang et al [46] studied individuals diagnosed with SAD to explore predictors of higher PEP levels. The results showed that higher PEP was predicted by self-attention, NA, social performance situations, and the use of safety behaviors. In addition, Badra et al [50] investigated PEP in a nonclinical sample of undergraduate students with high and low SA scores. Although no differences were detected between the groups, PEP was reduced to a single item, and no additional information on the social context was collected, which could have affected the results.

Another study that explored PEP showed that it decreased after a speech task [68]. The between-level average differed from the person-specific trajectories. This was the case not only for the decrease in PEP after the speech task but also in the temporal cascading relationship between PEP and the next measurement of PEP. The level of anxiety in the speech task predicted engagement in PEP, and this activated a more intense experience of the negative balanced memory, indicating the interconnectedness of cognitive-affective processes.

Bailey et al [51] studied perseverative cognition (worry and rumination) using physiological measures and found that there was a higher use of this type of repetitive thinking related to lower heart rate variability after negative social interactions. Finally, potential changes in momentary PEP throughout treatment were explored by Katz et al [41] showing that cognitive behavioral group therapy reduced levels of PEP.

In total, 3% (2/70) of the studies explored mind wandering, which is both an interesting and controversial topic. Traditionally, it was considered that mind wandering was just a negative process given that it was linked to the opposite of having a mindful disposition. However, the latest research has shown that it could be related to less boredom, more creativity, and a better mental health state [93].

In the study by Arch et al [49], the authors investigated differences in the frequency, range of content, and correlates of internal off-task thinking (ie, mind wandering). Relative to on-task thinking, internal off-task thinking was associated with worse mood, more self-focus, and less thought controllability for those with SAD compared to healthy controls. In addition, participants with SAD engaged in internal unrelated task thinking more frequently than those in the control group and presented more unintentional mind wandering on a trait questionnaire.

#### Specific Interactional Triggers, Patterns, and Activities

Geyer et al [62] investigated the association between NA in social interactions and perceived enjoyment of those interactions to explore specific real-time contributors to negative perceptions often experienced by individuals with SA. The results revealed that this association was more negative when SA was more severe, although the sample consisted of undiagnosed individuals.

Meanwhile, Blalock et al [15] studied the experience of flow in social and nonsocial situations in individuals with SAD and healthy participants. The results were contrary to the hypotheses, revealing that individuals with SAD presented flow more frequently in social situations than healthy participants. The authors explained this unexpected result using the concept of flow, which includes the component of experiencing a challenging situation as a defining feature. It is reasonable to think that people with SAD experience normal social interactions as more challenging than healthy participants.

In one study, the association between sexual activity and SA was explored in nonclinical individuals [71]. As could be expected, sexual activity was influenced by SA such that individuals with SA reported their sexual episodes as less pleasurable and reported being less connected with their partners as well as presenting a lower frequency of sexual activity. Overall, these results suggest that sexual activity is not fulfilling when experiencing SA.

Goodman et al [30] conducted 2 AA studies in which they concluded that, although individuals with SAD present fewer and less satisfying social relationships, they enjoy social interactions when they occur, which might indicate that they are happier with others than alone. In other words, experiencing SA and the relative concern about socializing does not hinder the pleasure of socializing.

These results are aligned with those of other 2 studies conducted on undergraduate students [54,83], which showed that SA was not related to a lower desire to be with others. However, in the study by Brown et al [54], a preference for solitude was found in interactions with unfamiliar people.

Villanueva et al [39] revealed that individuals with SAD presented a higher number of social interactions through their mobile phones than the control group. They were also the group with the least number of social interactions (vs the control group and individuals with depression). In-person interactions (ie, face-to-face) were revealed to be less related to increases in NA and decreases in PA.

In the study by Doorley et al [27], no significant association was found between the medium of communication (ie, digital vs face-to-face communication) and SA. That is, in both media, there was an association between SA and less positive and more negative emotions. Oren-Yagoda and Aderka [33] also explored media of communication in individuals diagnosed with SAD. In this case, the focus was on the media of communication and the associated perceptions and emotions. Individuals with SAD usually preferred to use voice and text media to a greater extent than visual media. However, the authors found that, despite this preference, when visual media were implemented, immediate increases in positive perceptions and emotions were experienced by people with SAD. These results support the idea that the selected medium functions as a safety behavior.

Meanwhile, Russell et al [44] revealed that individuals with SAD presented higher levels of submissive behavior and lower levels of dominant behavior compared to control participants. However, this was true in the presence of anxiety-eliciting cues, which means that there are certain situations that can be perceived as safe environments. In contexts of emotional security, all individuals with SAD presented an enhanced agreeable and deceased quarrelsome behavior, also meaning that there might be situations of security in which individuals with SAD can respond to positive appraisals with enhanced affiliative behavior.

Hur et al [66] found that individuals with SAD benefit more from having close friends, family members, and romantic partners in terms of resulting in lower levels of NA, anxiety, and depression compared to control participants. However, they tend to spend less time with those companions. These results emphasize the intact capacity of individuals with SAD to enhance their mood through social interactions.

In this line, Hannah Lee [65] obtained consistent results, suggesting a tight connection between the levels of SA and the degree of unfamiliarity and judgmentalness in the interactions. Namely, individuals with higher levels of SA presented a stronger association with the 2 processes as a consequence of being more sensitive to experiencing anxiety when facing interactions of the same level of unfamiliarity and judgmentalness compared to people with lower levels of trait SA.

Čolić et al [59] were the first to explore depersonalization and derealization in embarrassing situations. They showed that people with SAD presented more embarrassing social interactions than control participants, and as a result, they also presented more depersonalization and derealization, which can be seen as responses to strong emotions (including embarrassment) as well as attempts to cope with situational challenges.

#### Cognitive Factors

Social comparison is another important aspect to explore in individuals with SAD given that they usually tend to see themselves with a negative self-image [94,95]. In another study, Brown et al [54] showed that SA was associated with greater self-consciousness, which can also be aligned with the negative self-view that characterizes SAD.

Goodman et al [31] found that SA is related to less favorable and more unstable social comparisons, which can be explained by a negative self-image. Moreover, they demonstrated that, when people with SAD make less favorable social comparisons, they are especially fearful of others’ evaluations.

In a recent study, Brown et al [55] investigated interpersonal distress in heightened SA symptoms as predictors of suicidal ideation. Specifically, this study showed that hurdles to seeking social support and social comparisons mediated suicidal ideation.

Models of SAD have emphasized the central role of fear of evaluation in the appearance and maintenance of this clinical condition. This construct has been included in cognitive models related to attentional biases and negative interpretations of the self [96-98]. Although negative evaluation was considered a core dimension in early models of SAD, fear of positive evaluation has emerged as an important topic in recent years [78,99].

Another study explored anxiety sensitivity cognitive concerns and fear of negative evaluation as 2 potential predictors of SA amplification. Anxiety sensitivity cognitive concerns were shown to uniquely amplify arousal as a consequence of social stress, whereas fear of negative evaluation predicted anxiety fluctuations, indicating that these 2 cognitive constructs may be associated with SA in different ways [79].

By implementing 2 AA studies, Reichenberger et al [78] explored the interaction of both positive and negative evaluation, affect, and stress reactivity. Although the results were not fully in line with the hypotheses, fear of negative evaluation was negatively associated with PA. In addition, the results revealed that the closeness of the relationships was paramount to determine when the interaction was significant, with closer relationships being less anxiety provoking. Consistent with these results, positive and negative feedback seeking has been shown to be higher in individuals with SAD than in healthy participants [81], all of which is aligned with the mounting evidence developed in cross-sectional or laboratory settings. Similarly, Doorley et al [28] demonstrated that self-perceived intense positive events, which are normally reduced in SA, paradoxically provided more psychological benefits (reduced anxiety and motivation toward social situations as well as an increased sense of belonging).

Moreover, Nanamori et al [74] studied triggers of self-focused attention, which is another key component of classic cognitive models of SAD. The results showed that perception of gaze, evaluation, and authority predicted self-focused attention from the observer’s perspective, whereas perception of gaze also predicted self-focus on body sensation. Moreover, the perception of positive response and that of a stranger predicted self-focus on body sensation hinged on sex, suggesting that the positive response perception of female participants acted as a predictor of the self-focus on body sensation.

### Emerging Topics

#### Use of AA to Assess Psychological Interventions

Daniel et al [83], Katz et al [41], and Kivity and Huppert [72] implemented AA to explore the course of treatment. In the case of Katz et al [41], PEP, a putative maintenance factor of SA symptoms, was assessed over the course of a cognitive behavioral therapy intervention with a subset of the 60 included participants answering an AA. The intervention yielded significant reductions in both general and momentary PEP, and both types of PEP were significant predictors of SA severity after treatment.

Another study was conducted by Kivity and Huppert [72]. It explored how ER in individuals with high and low SA responded to a practice of cognitive reappraisal using self-report, laboratory tasks, and daily diaries. Although the group with high SA presented lower symptom severity and greater self-efficacy of reappraisal, daily anxiety was not significantly different.

Daniel et al [83] conducted a randomized controlled trial testing whether cognitive bias modification for interpretation could decrease negative interpretation bias. Both the active and control conditions received an AA throughout the treatment period (5 weeks). While the active group also received cognitive bias modification training, the control group only answered the AA. A total of 2 publications were identified from this study, reporting self-report and passive sensing outcome measures. Despite the interesting approach of incorporating multimodal assessment, both analyses yielded nonsignificant results.

#### Use of Sensors and Biosensors in AA

Over the last few years, new advancements in wearable sensors and biosensors have enabled us to incorporate them into AA studies. In the case of SA, this has led to a considerable body of evidence. Specifically, Bailey et al [51], Boukhechba et al [53], Chow et al [58], Daniel et al [82], Di Matteo et al [60], and Jacobson et al [47] conducted studies using sensors (GPS location and accelerometers) and biosensors (heart rate and heart rate variability).

Bailey et al [51] investigated perseverative cognition in relation to the parasympathetic nervous system, which is considered of utmost relevance in the regulation of stress and emotions [100]. In this study, individuals with both depression and SAD were monitored, and individuals with SAD presented the highest frequency of daily perseverative cognition, which was statistically associated with lower heart rate variability, moderated by negative social interactions. Jacobson and Bhattacharya [67] showed that spending time indoors was associated with anxiety and avoidance symptoms, and this association was significantly higher in individuals with SAD than in those with generalized anxiety disorder.

Boukhechba et al [53] and Chow et al [58] used GPS and Jacobson et al [47] used an accelerometer to demonstrate the capability of passive sensing to predict the severity of SA symptomology according to the level of activity. Moreover, Di Matteo et al [60] designed an app to capture ambient audio, GPS location, screen state, and light sensor data, and this app was shown to be able to identify SAD patterns of behavior in a relatively accurate way.

#### Exploring Idiographic Comorbidity Patterns

Although a vast array of studies included heterogeneous samples in terms of their diagnosis, only Piccirillo and Rodebaugh [77] had the objective of exploring SAD and major depressive disorder comorbidity, aiming to model person-specific trajectories of cognitive-affective and behavioral dimensions related to these disorders. By including only cisgender women with comorbid SAD and major depressive disorder, this study showed the utmost relevance in disentangling between-person, within-person, and person-specific patterns. For example, loneliness was revealed to be a common predictor of depressive mood and social avoidance at the group level; however, this was not the case when examining the idiographic networks.

#### Transcultural Differences

Only 1% (1/70) of the studies examined potential variations between different cultural groups. Lee et al [42] explored differences between European Americans and Asian Americans, showing that both groups experienced the same number of anxious events during social situations but Asian Americans presented more negative emotions in those moments.

## Discussion

### Principal Findings

Our review of 70 original studies using AA to explore SA showed that this methodology provides valuable real-time information on the momentary association of SA with several variables, such as context, affective dynamics, emotional states and regulation, social interactions, and other consequences and antecedents. This comprehensive understanding can contribute to better insights into the appearance and maintenance of symptoms and of this clinical disorder.

### Principal Themes Explored

Aligned with the burgeoning literature on AA, affect and emotional dynamics emerged as the most studied topics. These investigations revealed a trend of an increase in NA levels leading to a heightened experience of SA, as well as a growing attention to positive emotions and PA deficits in individuals with SAD. This review also supports the notion that negative emotions and affect are not enough to distinguish normal from pathological SA. As demonstrated by Park and Naragon-Gainey [45], AA may help shed light on the structural models of affect both between and within individuals’ variances, evidence that traditional cross-sectional research or long-term longitudinal research may not capture.

Most interestingly, most of the studies exploring affect trends explored them coupled with ER strategies, consistently extending the vast literature in this regard. Exacerbated NA and PA and dysfunctional strategies to cope with them form a dysfunctional pattern that may be responsible for the appearance and maintenance of SA and SAD [85]. The studies revealed that the interpersonal encounters of individuals with SAD may differ from those of controls in terms of ER use and type of ER strategy. Specifically, both intra- and interpersonal regulatory mechanisms have been shown to be associated with increasing levels of SA. In clinical populations, what was shown to influence the levels of SA was not the use of certain strategies but rather the lack of effectiveness of their use. However, this was not the case in healthy populations. Accordingly, a potential difference between SA symptoms in healthy and clinical populations may lie on the effectiveness or underuse of ER strategies.

In line with the mounting evidence exploring suppression and both experiential and behavioral avoidance in individuals with SAD, this systematic review showed coherent and robust results across the included studies concerning the maladaptive use of these 2 strategies. People with SAD may present an overreliance on the use of suppression and avoidance [85], resulting in a range of negative outcomes such as an increase in NA and a decrease in PA.

In contrast, cognitive reappraisal, a putatively effective strategy, does not yield straightforward results. The problem with individuals with SAD is more the ineffective use of cognitive reappraisal rather than the scarce use of this strategy, although the studies did not seem to coherently yield conclusive results.

Taken together, the results on affective dynamics and ER indicate how appropriate AA can be to study these processes, especially in the case of SA and SAD. AA may be particularly helpful in disentangling between- and within-person effects, which the literature demonstrates can have different or even contrary results, especially in the context of psychological interventions [101].

As a key takeaway message, AA shows that individuals with high SA symptoms or a diagnosis of SAD may not use a narrower repertoire of ER strategies but rather implement that repertoire with less skillfulness or less ability to identify when it is appropriate to implement a certain strategy. In this sense, it is essential to continue exploring the role of polyregulation and flexibility to identify which specific facet of ER contributes as a mechanism of action of SAD.

In that vein, substance use can be seen as a maladaptive behavior that functions as a behavioral strategy to regulate emotions and cope with situations that elicit symptoms of SA. This is particularly recurrent in SA-provoking situations, constituting a reinforcement cycle that operates similarly to other safety behaviors. In particular, alcohol consumption may function as a negative reinforcer, attenuating the negative self-perceived quality of interpersonal encounters and anxiety levels. When this occurs, alcohol use becomes a rapidly established maladaptive behavior with negative consequences.

In addition, maladaptive cognitions, emotional mechanisms, and behaviors were found to be activated when levels of anxiety increased. More specifically, cognitive aspects such as beliefs in capacity, effectiveness in regulating emotions, and the ability to differentiate emotions appear to be relevant in explaining how SA is activated.

As a general takeaway message, there is ample evidence showing the mutual directionality between cognitive and affective or emotional facets and behaviors in the appearance and maintenance of SAD. The several sections in which the studies were categorized constitute just one way of organizing the information. However, many of these studies can be understood as forms of cognition or ER or specific interactional patterns. A clear example is alcohol consumption, which is a behavior that, in the context of SAD, can be understood as an ER strategy.

In that sense, ER is currently a trending topic in AA, but it is important to integrate this increasing amount of knowledge into classic cognitive models. This is pertinent for psychopathological developments in general, and SAD is not an exception. Over the years, the most influential developments in SAD have been cognitive and behavioral models [96,98]. Paradoxically, in this review, cognitive processes remained an underexplored area. An example of this is mental imagery, which has been shown to be a transdiagnostic process that explains the appearance and maintenance of a range of clinical conditions, including SAD [94]; however, there is a dearth of studies on this crucial construct.

### Emerging Topics

An additional line of research that needs to be further explored is comorbidity to explore the mutual dependency of certain groups of signs and symptoms. However, given the lack of network analyses, this mutual dependency was not explored in depth. For example, there was only one study exploring suicidal ideation and attempts despite the ample existing literature on AA in suicide research [102] and the strong association between suicide and SA [103]. In the same vein, the relationship among personality, personality pathology, and SA is a relevant topic in contemporary psychopathology that could be further explored using AA strategies. Indeed, there is a growing body of evidence exploring personality and personality pathology dynamics and states, which should be considered in future studies of SA [9].

Another issue worth discussing revolves around the incorporation of AA into psychological interventions. This is an increasingly used practice and may be well integrated with routine outcome monitoring procedures that have been shown to yield significant effects when implemented in both controlled and naturalistic interventions [104]. Routine outcome monitoring is an increasingly implemented strategy that can connect research and practice in unprecedented ways. With that aim, it is necessary to create simple visualization interfaces to feed back the trajectories of certain patient variables. Some efforts have already been made in this direction [105]. If these endeavors are further developed, they can be used by clinicians as a clinical tool, and at the same time, researchers can collect naturalistic data.

Another topic with a lot of potential is the incorporation of behavioral and physiological processes by means of sensors and biosensors. Multimethod measurements that incorporate both passive and active assessments can be of tremendous relevance to harness the affordances of each approach. Together with the development of machine learning algorithms, the proliferation of EMIs is more plausible. This is very important to enhance the personalization of possible treatments.

Regarding data collection, there are now software solutions that are opening up unprecedented opportunities for future research. Older studies usually included PDAs or similar devices, which implies not only spending more resources to implement an AA study but also some degree of training in order for participants to be able to use these devices. Currently, there are studies that harness existing survey platforms such as *Qualtrics* to program either random or fixed prompts without the need for any specifically developed software.

Given that most of the studies were conducted in the United States and the rest were conducted in other Western high-income countries, the results should be generalized to other contexts. Cultural and contextual factors are determinant in all psychopathological conditions, and SAD is not an exception [106]. The proliferation of open-source platforms (eg, m-Path [107]) will permit the dissemination of this methodology to researchers without large budgets, such as researchers from low- and middle-income countries. This is crucial to guarantee that knowledge is not restricted to certain populations, fundamentally populations from Western, educated, industrialized, rich, and democratic countries. In addition, most of the studies (54/70, 77%) paid the participants to enhance the compliance rates, which turned out to be in line with the average compliance in AA literature (approximately 75% [108]). However, this should be considered in future research on AA that seeks to increase the external validity by means of ecological designs.

### Methodological Design

Regarding the methodological design of the studies included in this systematic review, there are important aspects to discuss. Most of the studies were well designed; advanced statistical strategies were applied; and, accordingly, most of this research was published in journals with a high impact factor. However, there is a methodological pitfall in AA research that revolves around the lack of psychometrically sound instruments, usually because of trying to reduce participant burden as much as possible. According to Hopwood et al [109], four aspects are essential when discussing the theoretical and methodological implications for AA research: (1) How should time be scaled? (2) How many assessments are needed? (3) How frequently should assessments be conducted? and (4) When should the assessments occur? Researchers using AA methods to conduct research on SA and SAD should carefully consider these questions both theoretically and empirically. Moreover, there is a wider consensus on the need to conduct more theory-driven hypothesis testing [110]. All these methodological aspects should be considered with caution together with the importance of increasing the transparency of reporting the results of AA research [111].

Power analysis was revealed as a weak methodological aspect, with many of the studies not calculating the required sample sizes to anticipate the number of needed participants. Potential limitations concerning the quality of the studies seem to be related to the lack of clear guidelines and standards, which have only recently started to emerge [112].

Regarding the data analysis, most of the studies used multilevel or hierarchical linear models [113]. Indeed, in cases in which ANOVAs or ordinary least squares models were used instead of hierarchical models, the results should be interpreted with more caution. They do not account for the dependency of the data, and in longitudinal assessments such as AA in which data are essentially nested, using these strategies may yield inaccurate pictures of the data [114]. In addition, AAs generally entail mounting random missing data, and multilevel mixed models are appropriate to deal with that data structure.

Future studies need to incorporate new modalities of data analysis that might provide more complex information to understand the dynamics of SA. For example, multilevel network analyses [115] would allow for shedding light not only on the nested structure of the symptoms but also on the interconnectedness at every moment of the individuals’ experiences and behaviors. In addition, new-generation time-series analyses such as the time-varying change point autoregressive models would allow for the detection of gradual and abrupt changes in SA markers over the course of the AAs [116]. Furthermore, the recently developed dynamic structural equation modeling method [117] is particularly suitable for intensive longitudinal data from AA. This method allows for a more accurate estimation of individual differences in means and autoregressive effects from AA data. Finally, machine learning strategies will be paramount not only to build predictive models that can better explain SA but also to implement EMIs based on people’s needs [118]. In the field of SAD, there is a dearth of studies on EMIs, which is surprising given the ample evidence that has been found using AA.

### Limitations

The results of this review should be considered in light of certain limitations. The first limitation concerns the inclusion criteria. Gray literature, including dissertations and preprint depositories, was not considered. Given the growing interest in this topic, we may have missed other relevant studies from these sources. However, this decision had the main aim of ensuring the rigor of including articles that had undergone a peer review process. In addition, we only included revised articles published in English, excluding articles published in different languages.

A second limitation is that this is the first synthesis that summarizes the literature on AA for SA, but further quantitative syntheses (ie, meta-analyses) should be conducted on the specific topics identified in this study. Thus, a qualitative review is a first step that contributes to taking stock of the principal topics studied in the field of SA and AA, but no conclusive statements should be drawn.

### Conclusions

This systematic review shows that AA constitutes a very powerful modality to grasp SA from a complementary perspective to laboratory experiments and usual self-report measures. Over the last few years, mounting research has been conducted showing important trends that are shedding light on the understanding of SA and SAD using this ecological tool that is revolutionizing the field.

## Appendix

Multimedia Appendix 1 Syntax used in database searches.

Multimedia Appendix 2 Characteristics of the included studies.

Multimedia Appendix 3 Social anxiety disorder ambulatory assessment research design overview.

Multimedia Appendix 4 PRISMA Checklist.

## Abbreviations

AA: ambulatory assessment
EMI: ecological momentary intervention
ER: emotion regulation
NA: negative affect
PA: positive affect
PEP: postevent processing
PRISMA: Preferred Reporting Items for Systematic Reviews and Meta-Analyses
SA: social anxiety
SAD: social anxiety disorder

## Data Availability

All data generated or analyzed during this study are included in this published article and its supplementary information files.
