# Supplementary material for: Studies of Social Anxiety Using Ambulatory Assessment: Systematic Review
Source: JMIR Ment Health. 2024 Apr 4;11:e46593. doi: 10.2196/46593 (PMC11027061; doi:10.2196/46593)
Supplement: Multimedia Appendix 1 [file mental_v11i1e46593_app1.docx]

## Appendix 1

**Syntax used in database searches:**

((ema) or ( "ecological momentary assessment" ) or ( "mobile health" ) or ( health ) or (smartphone) or ( esm ) or ( "experience sampling method" ) or ( "ambulatory assessment" ) or ( "personal digital assistant" ) or ( "ambulatory monitoring" ) or ( "real time data capture" ) or ( "real time monitoring" ) or ( "real time interventions" ) or ( "electronic diary" ) or ( "repeated observations") or ("diary data") or ("time series" ) ) and ( (glossophobia) or ( "public speaking anxiety") or ("public anxiety") or ("social anxiety" ) or ( "social phobia" ) or ( "fear of public speaking" ))
