# Supplementary material for: Studies of Social Anxiety Using Ambulatory Assessment: Systematic Review
Source: JMIR Ment Health. 2024 Apr 4;11:e46593. doi: 10.2196/46593 (PMC11027061; doi:10.2196/46593)
Supplement: Multimedia Appendix 2 [file mental_v11i1e46593_app2.docx]

**Multimedia Appendix 2**

Characteristics of the included studies.

| Study | N (Age; Gander) | Race/ Ethnicity | Comorbidity | Aim | Main findings |
| --- | --- | --- | --- | --- | --- |
| Arch et al [49] | 53 (25 SAD; 23.56; 65%F) | 78.43% White &  non-Latinx, 9.8%, Hispanic/Latinx, 5.88%, Asian American, 3.92%, Black/African  American and 3.92% Native American, including one multi-racial participant. | 24% of SAD individuals present a comorbid condition (GAD^a^, social phobia, or panic disorder) | To assess off-task and on-task thoughts | Participants with SAD engaged more in internally oriented off-task thinking but not externally oriented. Internally focused off-task thoughts had different content and mood correlates depending on whether clinical anxiety was present. In SAD, focusing on the activity at hand normalized thinking content and mood. |
| Badra et al [50] | 51 (25.3; 80.3%F) | Not specified | Not specified | To investigate whether the levels of cognitive bias are mediated by post-processing event. | Evaluating only one item of social rumination, no difference was found between individuals with high SA and low SA individuals. |
| Bailey et al [51] | 48 (16 SAD; 27.5; 56,2%F) | 18 Caucasians, 18 Asian Americans, 6 African Americans, and 6 Hispanic Americans | Not specified | To know the influence of social interactions on the use of perseverative cognition (PC) | In individuals with SAD, PC had a higher daily frequency which was associated with lower heart rate variability after negative social interactions. PC indicated a worsening mood. |
| Battista et al [52] | 132 (20.76; 24.24%F) | 85.5% White | Not specified | To investigate the relationship between SA and alcohol consumption | The consumption of alcoholic drinks was associated with a subsequent 4% decline in experienced SA. |
| Beltzer et al [23] | 114 (20.37; 74.6%F) | 72.8% White, 19.3% Asian, 7.9% African American, 2.6% Middle Eastern, and 2.6%  Native Hawaiian/Pacific Islander | Not specified | To test a contextual recommender algorithm for emotional regulation strategies. | The contextual algorithm was not the top-performing policy when strategies were classified into categories. When the top ten approaches were evaluated separately the contextual algorithm topped all other policies. |
| Blalock et al [14] | 79 (43 SAD; 28.9; 64.6 % F) | 54.4% Caucasian/White, 19% African American/Black, 12.7% Hispanic/Latino, 5.1% Asian American, 8.9% Other. | Not specified | To explore the interaction between SA and emotion regulation. | Individuals with SAD presented greater suppression and less cognitive reappraisal in daily life. |
| Blalock et al [15] | 73 (28.9; 65%F) | 54.4% Caucasian/White,19% African American/Black, 12.7% Hispanic/Latino, 5.1% Asian American, 8.9% Other | Not specified | To study the subjective experience of flow in SAD individuals. | SAD and healthy individuals presented equal frequency of flow. Social situations led to more flow in SAD individuals. |
| Boukhechba et al [53] | 228 (19.43; 62%F) | 52% White, 27% Asian, 5% black, 2% Latino, and 14% multiracial | Not clinical | (1) To understand the relationship between mobility and SA level, (2) To investigate whether mobility could predict SA scores. | Location entropy was negatively associated with SA (more pronounced on weekdays than on weekends). Was related to avoiding public areas and engaging in a lesser deal of leisure activities. |
| Brown et al [54] | 245 (19.5; 7.1%F) | 73% Caucasian and 27% African American | Not specified | To explore simultaneously social anhedonia and SA in daily life. | SA was associated with more negative affect. Greater self-consciousness and preference for solitude were found only in interactions with unfamiliar people. |
| Brown et al [55] | 211 (32.32; 81% F) | 73.0% White/European  American, 21.3% Hispanic/Latino, 11.4% Asian/  Pacific Islander, 7.6% Black/African American, 2.4% Arab/  Middle Eastern, and 1.9% other. | Not specified | To assess if daily cognitive, behavioral, and affective dimensions of interpersonal distress explained the relationship between  SA and suicidal ideation. | Initial levels of SA indirectly influenced the severity of suicidal ideation 1.5 months later. This influence occurred through unfavorable social comparisons and barriers to seeking support. No significant indirect effect of SA on suicidal ideation severity through loneliness. |
| Buckner et al [56] | 49 (19.1; 38.8%F) | 2.0% American Indian, 83.7% Caucasian, 2.0% Hispanic/Latino, 10.2% mixed, and 2.0% other | 51% of the total sample (not specified). | To deepen the relations among SA, cannabis craving, state anxiety, situational variables, and cannabis use. | Individuals with higher SA and craving were most likely to use cannabis. When others were using cannabis, those with elevations in both trait SA and state anxiety were more likely to use cannabis. |
| Buckner et al [57] | 51 (34.6; 33.3% F) | 86.3% White, 7.8% Black or African American, 3.9% “mixed,” and 2.0% Asian, with 2.0% endorsing His- panic/Latino ethnicity | Not specified | To examine the relation of SA to post-quit nicotine withdrawal severity following an unaided cessation attempt. | Increases in negative affect were linked to baseline SA. Increased SA is linked to more intense post-quit withdrawal symptoms and higher negative affect levels during a quit attempt. |
| Chow et al [58] | 63 (19.8; 51.4%F) | 42% white, 38% Asian, 4% black, 4% Latino, and 13% multiracial or unspecified | Not specified | To explore if the time spent at home is associated with negative and positive affect. | Higher SA, more negative affect, and less positive affect were associated with spending more time at home the following day. |
| Čolić et al [59] | 284 (47 SAD; 28.3; 66%F) | 100% Caucasian | Not specified | To investigate the duration and frequency of depersonalization and derealization in embarrassing social interactions in everyday life. | Individuals with SA experienced more embarrassing social interactions than controls and more depersonalization and derealization. |
| Daniel et al [24] | 124 (19.07; 66.9%) | 54.8% White, 30.6% Asian, 6.5% Black, 2.4% Hispanic/Latinx, and 2.4% Multiracial. | Not specified | To explore how different operationalizations of emotional regulation effectiveness led to diverse conclusions regarding which strategies are more effective. | A significant negative association between SA severity and effectiveness was observed, and engagement-oriented strategies were more effective than avoidance-oriented strategies. |
| Daniel et al [83] | 106 (20.44; 73.6%) | 67% White, 16% Asian, 6.6% Black, 1.9% Middle Eastern, 8.5% Multiracial | Not specified | To investigate if state measures in treatment are sensitive to an online intervention effect in daily life. | Trait negative interpretation bias was reduced by the online training. So did trait and state of SA symptoms and fear of negative evaluation. These changes were not unique to the group that received the intervention. |
| Daniel et al [82] | 98 (20.50; 74.74% F) | White (66.3%); Asian (17.3%); Black (6.1%); Middle Eastern (1.02%); Multiracial (8.1%) | Not Specified | To evaluate the influence of an online training intervention concentrating on the interpretive threat through GPS data. | No reliable association has been found between the intervention and passively collected mobility data. |
| Daniel et al [22] | Low-social-anxiety group: 42 (19.38; 71.43%F)  High-social-anxiety group: 113 (20.38; 71.43%F) | Not specified | Not specified | To investigate the relationship between emotion regulation strategies and SA severity (high or low). | Increased diversity in regulating emotions within avoidance-focused strategies was associated with a higher chance of being in the more severe group. Greater diversity in all emotion regulation approaches, both within and between avoidance and approach strategies, was linked to increased severity of SA. Diversity within avoidance-oriented strategies showed a significant correlation across both trait and state levels. |
| Daros et al [25] | 115 (19.09; 66.09% F) | White (55.6%), Asian (26.09%), Black (5.2%), Multiracial (3.4%), Latinx/Hispanic (2.6%), and other (6.09%) | Not specified | To assess how actual and desired social situations predicted reports of emotion regulation strategy. | In highly negative affect states, SA predicted the use of emotion regulation strategies. Social desire predicted the use of any type of emotion regulation strategy. This interaction remained not significant when considering social situations. |
| Di Matteo et al [60] | 84 (28.8; 42%F) | Not specified | Not specified | To predict GAD, SAD, and depression using data extraction through a mobile application. | Ambient audio, GPS location, screen state, and light sensor data were able to predict features of SAD and depression but not GAD. |
| Doorley et al [28] | 125 (26.39; 50,4%F) | 53.2% White, 16.1% Asian, 12.9% Black, 11.3% multiracial/other, and 6.5% Hispanic. | Not specified | To examine the impact of trait SA on momentary changes in emotions, sense of belonging, and social approach versus avoidance motivation after positive events. | High SA was significantly associated with elevated anxiety, social avoidance motivation, lower momentary happiness, and sense of belonging. Elevated SA benefited more from self-perceived intense positive events. |
| Doorley et al [27] | Study 1 (S1): 125 (19.3; 50.4%F)  Study 2 (S2): 303 (31.1;66%F) | S1: 53.2% White, 16.1% Asian, 12.9% Black, 11.3% multiracial/other, and 6.5% Hispanic.  S2: 47.1% White, 20% Asian, 14.2% Black, 8.4% Hispanic, 3.5% Middle Eastern, 6.8% Other. | Not specified | To understand the feelings and behaviours of people with high SA interacting digitally versus face-to-face. | Regardless of the medium of communication, no differences were found in the likelihood of engaging in conversation in the presence of SA but were observed an increase in negative emotions. The association between SA and emotions is not mediated by the timing of communication. |
| Farmer and Kashdan [61] | 89 (21.7; 81%F) | 50 White/European, 11 Asian/Asian American, 9 Black/African American, 9 Hispanic, 9 Middle Eastern, 1 Native American, and 7 others | Not specified | To demonstrate that individuals with higher levels of social anxiety report significantly less intense positive affect in their daily lives | High SA was related to positive emotion suppression, fewer positive social events, and less positive emotion on the subsequent day. Low SA was associated with fewer negative social events on the following days in which cognitive reappraisal had been used in order to reduce distress. The use of cognitive reappraisal did not lead to any change in people with high SA. |
| Farmer and Kashdan [16] | 79 (40 SAD; 28.86; 64.5%F) | 54.4% Caucasian/ White, 19% African American/Black, 12.7% Hispanic/ Latino, 5.1% Asian/Asian American and  8.9% others | 26 SAD individuals present a comorbid condition (18 anxiety disorder; 7 MDD^c^, and 1 bipolar disorder) | To compare the daily affective and self-esteem instability between people with SAD and healthy adults. | The SAD group showed difficulties in self-regulation as well as greater instability and a tendency to extreme shifts in both affective and self-esteem. |
| Geyer et al [62] | 60 (19.9; 55%F) | 40% White, 5% Black, 5% Hispanic, 37% Asian, 8% Mixed, and 5% unknown | Not specified | To explore if affect judgments during social interactions affect later perceptions of such interactions and if this varies depending on the intensity of SA and depression. | The association between negative affective in social interactions and enjoyment of those interactions is greater according to the levels of SA severity. |
| Goodman et al [63] | 160 (21.7; 75.3%F) | 59.1% White/European American, 9.7% Black/ African American, 9.7% Asian/Asian American, 9.1% Hispanic/Hispanic American, 5.8% Middle Eastern, .6% Native American, and 5.2% other. | Not clinical | To investigate the relationship between SA and alcohol consumption | Alcohol consumption moderated the significant negative association between SA and a range of healthy social interactions. |
| Goodman et al [17] | 84 (41 SAD; 28.68; 65%F) | 54.8% Caucasian, 20.2% African American, 9.5% Latino/Hispanic | 11 social phobia, 7 MDD, 5 dysthymia, 5 PTSD^f^, 3 GAD, 2 panic disorder, 2 OCD^d^, 1 agoraphobia | To explore goal pursuit content, motives, consequences, and daily correlates of strivings. | Individuals with SAD showed greater difficulties in pursuing striving and more efforts to control, manage, and get rid of emotions. |
| Goodman et al [18] | 73 (36 SAD;  29.26; 65.8%F) | 52.1% Caucasian/ White, 21.9% African American/Black, 11.0% Hispanic/ Latino, 4.1% Asian/Asian American, 1.4% Middle Eastern and 9.6% other. | Not specified | To explore whether emotion beliefs predict the use of emotion regulation. | Among participants diagnosed with SAD, higher emotion control values and lower emotion malleability beliefs were found. In both groups, suppression was positively associated with emotion control but negatively associated with malleability, which was positively associated with reappraisal. |
| Goodman et al [32] | 86 (41 SAD; 30.37; 61,6% W) | 48.8% White/Caucasian; 18.6% Black/African American; 14.0% Asian/Pacific Islander; 7.0% Latin/Hispanic; 2.3% Arab/Middle Eastern; 9.3% other | In the SAD group: 11 MDD; 1 Persistent Depressive disorder; 9 GAD; 7 Alcohol use disorder; 6 PTSD; 3 panic disorder and 2 agoraphobia. | To examine differences in the appraisal of contextual demands and emotional regulation in people with and without SAD | Disengagement strategies and inflexibility were mainly observed in SAD patients. Regulation patterns between the two groups have been shown to be similar, especially in engagement strategies. |
| Goodman et al [30] | S1: 87 (42 SAD: 30,3; 62%F)  S2: 77 (39 SAD; 28.8; 63,6% W) | S1: 48.3% White; 19.5% Black/African American; 13.8% Asian/Pacific Islander; 6.9% Latino/Hispanic; 2.3% Arab/Middle Eastern, and 9.2% other.  S2: 51.9% White; 23.4% Black/African American; 3.9% Asian/Asian-American; 10.4% Hispanic/Latino/Mexican American; 1.3% Middle Eastern, and 9.1% other. | S1: In SAD group: 21% GAD; 7% panic disorder; 5% agoraphobia; 31% MDD; 17% alcohol use disorder; and 14% PTSD.  S2: In SAD group: 8% GAD; 5% panic disorder; 3% agoraphobia; 28% MDD; 5% substance disorder; 13% PTSD. | S1: To compare positive and negative affect in people with SAD when alone versus when with others.  S2: To examine the emotional experiences according to the interactions. | SAD patients reported lower positive affect and higher negative affect in both social and non-social situations, suggesting lower rewards in socialization. Both groups reported higher positive affect when with others. |
| Goodman et al [31] | S1: 186 (24.04; 71,5% F)  S2: 85 (42 SAD; 30.3; 62% F) | S1: 57.8% White; 12.7% Latino/Hispanic; 12.1% Asian; 7.5% African American; 1.7% Middle Eastern; 1.2% Native American, and 6.9% other.  S2: 48.3% White; 19.5% Black/African American; 13.8% Asian/Pacific Islander; 6.9% Latino/Hispanic; 2.3% Arab/Middle Eastern, and 9.2% other. | S1: Not specified.  S2: In the SAD group: 11 MDD; 2 persistent depressive disorder; 9 GAD, 7 alcohol use disorder; 6 PTSD; 3 panic disorder; 2 agoraphobia. | To explore the nature of social comparisons made in everyday life, and their links to immediate emotion and SA. | Higher social comparisons and momentary affect were observed in people with high traits of SA. Participants generally increased favourable comparisons in the company of others. |
| Goodman et al [29] | 85 (41 SAD; 29; 65%F) | 52.6% Caucasian, 21.8% African American, 10.3% Latino/Hispanic, 3.8% Asian/American, 1.3% Middle Eastern, and 10.3% other | In SAD group: 6 MDD; 3 GAD; 2 OCD; 1 panic disorder; 1 agoraphobia | To evaluate how far patients with SAD saw anxiety and discomfort as a barrier to pursuing personal goals and finding purpose in their lives. | In patients diagnosed with SAD, both anxiety and pain were found to be interfering factors in goal attainment and an inverse association between daily meaning in life and perceived emotion-related goal interference. |
| Goodman et al [64] | 54 (26 SAD; 39.94; 61.1%F) | 51.9% Caucasian, 16.7% African American, 9.3% Latino/Hispanic, 3.8% Asian/Pacific Islander, 13% Arab/Middle Eastern1.9%, and 7.4% other | In SAD group: 7 MDD; 5 Alcohol use disorder; 6 GAD; 4 PTSD; 2 panic disorder; 1agoraphobia; 1 Persistent Depressive Disorder; 1 Substance Abuse Disorder; 1 OCD | To determine if participants with SAD differed from controls (1) in affiliation and coping motives, (2) in their likelihood of experiencing positive drinking consequences, and (2) how affiliation and coping motives predicted positive drinking consequences. | The SAD group was more likely to believe that drinking reduced their anxiety. Both groups had similar experiences of positive social drinking outcomes and found that, on days when they were motivated to drink to enhance social interactions or cope with distress, they were more likely to experience positive consequences from drinking. Participants with SAD reported stronger motives for drinking related to anxiety coping, SA coping, and depression coping. |
| Hannah Lee [65] | 92 (22.88; 54%F) | Not reported | Not reported | To explore how state SA is related to the characteristics of people someone interacts with and how this connection might vary depending on their trait of social anxiety. | Perceived judgmentalness and unfamiliarity of interaction partners were positively linked to state SA. These associations were more pronounced in individuals with high trait SA. There were uncovered group differences in how various types of interaction partners and social settings were related to state SA, including factors like the relationship with the partner (e.g., acquaintance vs. close friend/romantic partner) and the context of the interaction (e.g., authority vs. work/school). |
| Helbig-Lang et al [46] | 49 (39.6; 64%F) | Not specified | 47% of the sample (15 affective disorders; 11 specific phobias; 3 agoraphobia) | To examine Post-Event Processing (PEP) in real moments after social situations. | Several variables such as self-attention, negative affect, and safety behaviour use predicted PEP. |
| Hur et al [66] | 228 (18.8; 51.3%F) | 62.7% White, 17.5% Asian, 8.3% African American, 4.9% Hispanic, 6.6% Multiracial/Other | Not clinical | To identify the situational factors associated with the real-world experience of SA. | At baseline, high SAs compared with low SAs reported lower self-efficacy of reappraisal and higher frequency and self-efficacy of suppression, but no differences emerged in the reappraisal task. Following the intervention, the HSA-R compared with the HSA-M reported lower symptom severity, greater self-efficacy of reappraisal but equal daily anxiety. HSA-R used reappraisal mostly combined with suppression (74.76% of situations). Post hoc analyses demonstrated that clinical diagnosis, but not severity, moderated the intervention effect. |
| Jacobson et al [47] | 72 (19.8; 51%F) | 41% white, 37% Asian, 4% black, 4% Latinx/Hispanic, 12% multiracial or unspecified | Not clinical | To test the utility of passive smartphone sensor data gathered over 2 weeks as predictors of SAD symptoms in healthy subjects. | Smartphone sensor data could accurately detect SA symptom severity and discriminate SA symptom severity from depressive symptoms, negative affect, and positive affect. Less socially anxious individuals showed greater oscillatory frequency than high SA counterparts |
| Jacobson and Bhattacharya [67] | 32 (19.56; 50%F) | 65.62% White/Caucasian, 3.12% Black/African American, 12.50% Hispanic/ Latina/Latino, 12.50% Asian/Asian American, 3.12% Multiracial/ Multiethnic, and 3.12% other | Not specified | To forecast forthcoming anxiety symptoms in individuals who have reported symptoms of clinical anxiety disorder. | Deep learning models could predict the majority of total variation in anxiety symptoms and predicting a large proportion of within-person variation at the hour-by-hour level. |
| Kane and Ashbaugh [68] | 101 (20.13; 64 F) | 38.6% Caucasian/White; 13.9% Asian; 13.9% African Canadian/Black; 9.9% Middle Eastern; 3% Hispanic; 2% Other; 1% European, 1% Indian; 16.9% multiple ethnicities | 15.8% Depressive disorders; 16.8% anxiety disorders; 2.0% SAD. | To examine (1) the time courses and predictors of post-event and anticipatory processing, (2) a possible cascade of negative thinking and anxiety (3) post-event processing and possible changes, and (4) phenomenological memory qualities | Both post-event and anticipatory processing declined. Greater anxiety during the speech and less favorable performance assessments were linked to increased post-event processing. Changes in post-event processing were not connected to shifts in performance assessments over time. Higher post-event processing the day after the first speech was linked to greater anticipatory processing before the second speech. Participants who engaged in more post-event processing also remembered the first speech differently, perceiving it as more negative and emotionally intense. |
| Kashdan and Steger [69] | 97 (19.7; 66%F) | Not reported | Not clinical | The influence of SA and struggles with the self-regulation of emotion during everyday life to understand whether, when, and why socially anxious individuals experience diminished hedonic activity | Trait SA was related to less positive affect and fewer positive events in everyday life. Days of elevated SA led to fewer positive emotions and an increase in suppression of emotions. Regardless of trait SA, participants reported the most intense positive emotions when SA was low, and acceptance was more used. |
| Kashdan and Collins [70] | 38 (26.9; 47.4%F) | European-American 81.6%. | Not clinical | To study the positive and angry emotional states related to the SA trait in each moment of their everyday environment. | Time spent feeling angry increased SA and decreased time spent feeling relaxed and happy. However, happiness increased in the company of others. Overall, those with higher levels of anxiety reported greater episodes of anger in both social and non-social situations. |
| Kashdan et al [71] | 150 (23.4; 77.3%F) | Not reported | Not clinical | To examine the relationship between SA and contact activity (frequency and quality) | Relationship between SA and frequency of sexual contact was significant in women but not in men. Low SA was related to an increase of feelings of connectedness in close and intimate relationships. Results of SA were not influenced by levels of depressive symptomatology. |
| Kashdan et al [20] | 76 (38 SAD; 27.9; 63.2% F) | 50% Caucasian, 18.4% African American, 13.2% Latino/Hispanic, 5.3% Asian, 2.6% Middle Eastern, 10.5% other | 21.1% MDD; 13.2% dysthymia; 7.9% panic disorder; 13.2% PTSD; 5.3% GAD; 5.3% OCD; 28.9% specific phobia. | To examine the association between experiential avoidance and positive emotions. | SAD individuals presented less intense positive emotions and more experiential avoidance compared to healthy individuals. However, there were no differences in self-control depletion between groups. |
| Kashdan et al [21] | 75 (37 SAD; 27.9; 63.2% F) | 50% Caucasian, 18.4% African American, 13.2% Latino/Hispanic, 5.3% Asian, 2.6% Middle Eastern, 10.5% other | In the SAD group: 11 (7 specific phobia MDD; 5 PTSD; 2 GAD; 2 OCD; 2 agoraphobia) | To examine the effect of experiential avoidance on SA y in everyday life. | When it came to anxiety feelings during social encounters, avoidance was positively correlated with them, and this association was stronger in people with SAD. |
| Kashdan and Farmer [19] | 85 (43 SAD; 28.5 61,6%F) | 46 White/Caucasian, 17 Black/African American, 9 Hispanic/Latino, 4 Asian/Asian American, 1 Middle Eastern, and 8 other. | Of the SAD group: 19 other anxiety disorders, 8 MDD or dysthymia and 1 bipolar disorder. | To investigate the emotion differentiation in daily life and if these differences translate to impairment in social functioning | SAD individuals presented less negative (but not positive) emotion differentiation during random prompts and social interactions. These differences were not explained by emotion intensity or variability. |
| Katz et al [41] | 60 (34.62; 40% F) | 88.3% white, 3.3% Asian, 1.7% Hispanic and 6.7% other | Not reported | To examine how momentary and general PEP change over the course of treatment, and how such changes predict treatment outcome | Momentary PEP decreased over the course of treatment, which predicted lower SA symptomatology after treatment. |
| Kivity and Huppert [72] | 124 (83 SAD; 24; 65.1%F) | Not reported | Not reported | To examine ER among individuals with high and low SA and the effects of 1 week of practiced cognitive reappraisal using self-report, daily diary measures and lab tasks | At baseline, high SA individuals compared with low SA individuals presented lower self-efficacy of reappraisal and higher frequency and self-efficacy of suppression. After the intervention, the high SA group reported lower symptom severity, greater self-efficacy of reappraisal but equal daily anxiety. High SA individuals used reappraisal mostly combined with suppression. |
| Kim and Kwon [73] | 119 (66 SAD; 22.20; 67.2%) | Not reported | In SAD group: 4 MDD; 1 Unspecified depressive disorder; 10 GAD; 8 social phobia; 1 Alcohol abuse; 1 Alcohol dependence; 1 Bulimia nervosa. | To examine how maladaptive emotion regulation strategies, affect negative emotions in predicting drinking among individuals with SAD. | Rumination might be an important risk factor when experiencing negative emotions, especially for the SAD group. |
| Ladis et al [26] | 128 (19.1; 65.6%F) | 54.6% White, 29.6% Asian, 6.25% Black, 2.3% Hispanic/Latinx, 3.1% Multiracial 3.9 Other | Not specified | To investigate who engages in polyregulation, the timing of polyregulation use, and the effectiveness of polyregulation when employed. | Participants reported using various strategies to manage their emotions. They were more likely to polyregulation when experiencing strong negative emotions and when motivated to change their feelings. Other factors like sex, mental health symptoms, social context, and the effectiveness of their strategies were not linked to polyregulation, and the intensity of their current emotions didn't change these patterns. |
| Lee et al [42] | 83 (20; 80,7% F) | 45 Asian American; 38 European American | Not reported | To study if Asian American university students experienced more SA than European Americans. | Both groups reported a similar number of events that evoked anxiety in social situations. However, Asian Americans presented more negative emotions. |
| Nanamori et al [74] | 22 (28.82; 18 F) | 100% Asian | Not reported | To identify (1) the factors that elicit self-focused attention in social situations, and (2) the triggers that increase self-focused attention. | Gaze, evaluation, and authority perceptions predicted observer-perspective self-focused attention. Gaze perception also influenced self-focus on body sensation. Controlling for fear, gaze perception predicted both self-focused attention, and authority perception predicted observer-perspective self-focused attention. Fear of evaluation, but not perception of evaluation, impacted observer-perspective self-focused attention. |
| Naragon-Gainey [43] | 135 (30.4; 72%F) | 70% White, 14% Black, 11% from any Asian ethnicity, 9% Hispanic, 2% American Indian | Not reported | To assess momentary affect and symptoms, items were selected from the PANAS^e^ and IDAS^b^. | While between-person variance in negative affect and concurrent levels of negative affect predicted SA, positive affect did not. Furthermore, neither positive nor negative affect significantly predicted subsequent SA symptoms in prospective, within-person analyses. |
| O’Grady et al [75] | 476 (18.73; 249F) | 86% White, 6% Asian/Pacific Islander, 4% Black/African American, 3% Latino/Hispanic and 1% other. | Not clinical | To study the relationship between SA and alcohol use. | High SA individuals drank independently from embarrassing events, while low SA individuals reduced their drinking showing an adaptive response. |
| Oren-Yagoda et al [35] | 88 (44 SAD, 28; 50% W) | Not reported | In the SAD group: 43.2% MDD; 9.1% agoraphobia; 4.5% panic disorder; 4.5% OCD; 11.9% GAD.  In no SAD group: 2.3% GAD. | To examine envy in SAD: levels, contexts and role in maintaining or elevating anxiety. | Higher levels of envy were found among SAD patients, especially in social contexts and in visual communications rather than in voice or text communications. Subsequent anxiety was predicted by envy. |
| Oren-Yagoda and Aderka [33] | 88 (44 SAD, 28.9; 50% W) | All participants self-identified as White/Middle Eastern | In the SAD group: 43.2% MDD; 9.1% agoraphobia; 4.5% panic disorder; 4.5% OCD; 11.9% GAD.  In no SAD group: 2.3% GAD. | To examine the use of different communication mediums and their immediate effects on perceptions and emotions in individuals diagnosed with SAD | SAD group leaned more towards utilizing voice/text channels and less visual platforms. Engaging with visual platforms led to immediate boosts in positive perceptions and emotions for individuals with SAD. |
| Oren-Yagoda et al [36] | 88 (44 SAD, 28.9; 50% W) | Not reported | In the SAD group: 43.2% MDD; 9.1% agoraphobia; 4.5% panic disorder; 4.5% OCD; 11.9% GAD.  In no SAD group: 2.3% GAD. | To examine loneliness in SAD | Individuals with SAD experience elevated loneliness following social interactions, and there is a reciprocal relationship between anxiety and loneliness. The characteristics of social situations, such as negativity, positivity, and meaningfulness, impact the experience of loneliness for individuals with SAD. |
| Oren-Yagoda et al [34] | 88 (44 SAD, 28.9; 50% W) | Not reported | In the SAD group: 43.2% MDD; 9.1% agoraphobia; 4.5% panic disorder; 4.5% OCD; 11.9% GAD.  In no SAD group: 2.3% GAD. | To investigate variations in the experience of pride among individuals with and without SAD. | Individuals with SAD experience lower levels of pride. Situations perceived as highly negative and positive, or as highly meaningful and positive, are linked to the highest levels of pride. The experience of pride is associated with subsequent reductions in anxiety among individuals with SAD. |
| O’Toole et al [37] | 164 (22.4; 64%F) | Not reported | Not clinical | The potential difference emotion differentiation in low and high SA and the differences in choice of ER strategies depending on intensity levels of emotions. | High SA and poor negative emotion differentiation presented the least use of cognitive reappraisal. High SA individuals used more suppression strategies despite the ability to differentiate positive emotions. |
| O’Toole et al [38] | 164 (22.4; 64%F) | Not reported | Not clinical | The potential difference in emotion regulation flexibility in low and high SA and the differences in choice of emotion regulation strategies depending on intensity levels of emotions. | SA moderated the relationship between emotion intensity (positive association) and experiential avoidance. Specific emotions (guilt, nervousness and sadness) significantly showed to increase levels of experiential avoidance. |
| Papp et al [76] | 297 (19.5; 69%F) | Not reported | Not reported | To explore the moderating role of SA on the link between negative mood and prescription drug misuse in daily life. | Individuals with higher levels of SA showed a stronger connection between momentary negative mood and prescription misuse. |
| Park and Naragon-Gainey [45] | 129 (43,4% SAD; 30.4; 71.3% F) | 70.5% Caucasian, 14.7% African American, 9.3% Hispanic/Latino, 7.8% Asian, 2.3% American Indian/Alaska Native, 2.3% Middle Eastern (multiple categories possible) | Not reported | Explore the association of emotional clarity with internalizing symptoms (i.e., depression, SA, panic, and worry). | Greater SA symptoms predicted higher levels of emotional clarity through unsuccessful emotion regulation. |
| Piccirillo and Robedaugh [77] | 35 (21.37; 100%F) | 51.43% White; 28.57% East Asian; 17.14% Black and 5.71% Hispanic | 34.29% panic disorder; agoraphobia 22.86%; 22.86% GAD and 22.86% alcohol use disorder. | To compare   group and person-specific models of SAD-MDD | Multilevel and person-specific network analyses revealed between-group, within-group, and individual-specific patterns |
| Reichenberger et al [78] | S1: 50 (23.6; 66%F)  S2: 59 (39.9; 78%F) | Not reported | Not clinical | To correlate daily correlates of negative and positive fear of evaluation, affect and different types of stressors. | In study 2 but not in study 1, fear of negative evaluations predicted lower positive affect. In both studies negative affcet reactivity to stressors emanating from the distant social network was increased in individuals high in fear of negative evaluations or fear of positive evaluation. |
| Rinner et al [40] | 284 (47 SAD; 28.3; 66%F) | 100% caucasian | Not specified | To investigate the memory experience gap of sadness, SA, happiness, and physical activity for participants diagnosed with MDD, SAD and participants without such diagnoses. | Results indicate significant differences in the Memory– experience gap with respect to the experience that was salient to them (e.g., MDD group – sadness; social phobia group – social anxiety; control group – happiness). |
| Russell et al [44] | 80 (SAD = 40; 29.23; 50%F) | Not reported | 1 dysthymia; 1 antisocial personality; 1 panic disorder; 4 agoraphobia; 5 OCD; 1 PTSD; 11 GAD. | To explore the influence of situational anxiety and emotional security on interpersonal behaviors. | Individuals with SAD reported higher levels of submissive behavior and lower levels of dominant behavior in comparison to controls. |
| Saulnier et al [79] | 83 (29.66; 59% F) | 77.1% White, 3.6% Black, 12.0% Asian, 7.2% other or multiple races. 3.6% Hispanic. | 22.9% anxiety disorder; 8.4% SAD | To examine the impact of Anxiety sensitivity social concerns (ASSC) and fear of negative evaluation (FNE) on anxious arousal and anxious apprehension following stressors. | ASSC was associated with increased anxious arousal when there were social stressors present. FNE was linked to higher anxiety levels following nonsocial stressors. |
| Seah et al [48] | S1: 54 (31 SAD; 32; 75,9% F)  S2: 193 (20,07; 79,3% F) | S1: White/European 87,03%; Black/African 7,4%; Asian 3,7%  S2: White/European 81,3%; Black/African 9,8%; Asian 2,6%; American Indian/ Alaska native 0,5%; Biracial/Multiracial 4,7%; Other 1,0% | S1: 59% MDD in the SAD group.  S2: Not clinical | To investigate whether the positive association between rumination and social avoidance would be moderated by negative emotion differentiation (NED) as a protective factor. | Both avoidance and rumination were found to be significantly moderated by low NED. |
| Villanueva et al [39] | 284 (47 SAD; 28.3; 66%F) | 100% caucasian | Not specified | To examine if technological social interactions are used equally as often as face-to-face social interactions. As well as if those potential differences are related to unpleasant emotions. | SAD engaged more often in phone social interactions in comparison to control healthy non-clinical individuals, who in turn engaged more often in face-to-face social interactions. A positive relationship between negative affect and the frequency of technological social interactions, and a positive relationship between positive affect and the frequency of face-to- face social interactions was detected. |
| Walukevich-Dienst et al [80] | 257 (21.8; 51% F) | 49.4% non-Hispanic White, 14.8% Hispanic/Latinx, 17.1% Asian/South Asian, 5.8% Black/African American, 8.9% more than one race, and 18.8% another race. | Not specified | To test whether using coping mechanisms for SA was linked to increased alcohol and cannabis consumption, as well as their respective consequences. | Days when people used coping strategies for SA were associated with increased alcohol and cannabis consumption, along with their respective consequences. These effects were consistent regardless of an individual's baseline SA symptoms. |
| Wilson et al [81] | 61 (21 SAD; 26.9; 75%F) | Asian 42.62% and White 39.34% | Not specified | To investigate differences in the frequency, topics, and targets of positive and negative feedback seeking. | No significant differences were found between the SAD group and the healthy control with respect to seeking positive, negative, or general feedback. |

^a^GAD: generalised anxiety disorder

^b^IDAS: Inventory of Depression and Anxiety Symptoms

^c^MDD: major depressive disorder

^d^OCD: obsessive-compulsive disorder

^e^PANAS: Positive and Negative Affect Schedule

^f^PTSD: posttraumatic stress disorder
