# Supplementary material for: Studies of Social Anxiety Using Ambulatory Assessment: Systematic Review
Source: JMIR Ment Health. 2024 Apr 4;11:e46593. doi: 10.2196/46593 (PMC11027061; doi:10.2196/46593)
Supplement: Multimedia Appendix 3 [file mental_v11i1e46593_app3.docx]

**Multimedia Appendix 3**

SAD-AA Research design overview.

| Study | Screening - SAD symptoms | SAD - AA | Other AA variables | Software or pompt delivery | Hardware | Observations | Data analysis |
| --- | --- | --- | --- | --- | --- | --- | --- |
| Arch et al [49] | DSM-5 – ADIS^a^ | No | Off-task thinking  Attention directed outwards/inwards  Negativity/Positivity thought content  Mood  Self-focused thought content  Thought controllability  Though bothersomeness | Qualitrics | Participant’s smartphones | 8/5 (40) | MLM^i^ |
| Badra et al [50] | SPIN^q^ | No | Social rumination | movisensXS | Motorola G8 if they did not have a smartphone | 5/4 (20) | MLM |
| Bailey et al [51] | DSM-IV-TR or MINI^g^ | No | Heart Rate Variability, Mind Wandering  Perseverative cognition  Sleep | https:// opendatakit.org | Bodyguard 2 (Firstbeat) | Not specified | MLM |
| Battista et al [52] | SIAS^o^ | State  SA | Alcohol consumption | e-mail reminders | Palmtop computers (Dell Axim X51) | 6/22 (132) | MLM |
| Beltzer et al [23] | SIAS | No | Emotional regulation strategies  Cognitive reappraisal | MetricWire | Participant’s smartphones | 7/6 (35) | ANOVA |
| Blalock et al [14] | ADIS and DSM IV | No | Emotional experience  Emotion regulation | Self-applied | - | 1/14 (14) | MLM |
| Blalock et al [15] | SCID-I/ND^m^ | No | Small battery of questions about their current experience | PMAT^k^ | Palm Pilot Z22 | 8/7 (56) | MLM |
| Boukhechba et al [53] | SIAS | No | SIAS Score | OpenStreetMap (OSM) | Participant`s smartphones | 159 seconds /14 | MLM |
| Brown et al [54] | SPS^r^ | No | Affect; social contact; cognitions; activities | iESP software | Palm Pilot Zire | 6/14 | MLM |
| Brown et al [55] | SIAS | No | Social support seeking; Barriers to seeking support; Unfavorable social comparisons; Loneliness | MetricWire | Participant`s smartphones | 1/14 | X |
| Buckner et al [56] | ADIS-IV-L , SIAS | State anxiety | Craving  Situation type/cannabis use  Self-cannabis use | Satellite Forms 5.2 developed by Pumatech | Palm® (Z22 Handheld) | 3/14 | MLM |
| Buckner et al [57] | IDAS^d^ | Not specified | Not specified | - | PalmPilot | 3/14 | MLM |
| Chow et al [58] | SIAS | No | positive affect and negative affect.  Location | Sensus | Participant’s smartphones | 6/14 | MLM |
| Čolić et al [59] | SCID^l^ | No | Social interactions  Depersonalization  Derealization | - | Smartphone provided by researchers | 5/7 | MLM |
| Daniel et al [24] | SIAS | No | Positive and negative affect.  Emotion regulation strategies | Sensus | Participant’s smartphones | 6/14 | MLM |
| Daniel et al [83] | SIAS | Single ítem of SA | Ability to rate cognitive reappraisal,  Social avoidance  Fear of avoidance emotion regulation strategies | MetricWire | Participant’s smartphones | 6/35 | LMM^e^ |
| Daniel et al [82] | SIAS | No | Cognitive bias modification for interpretations (CBM -I) | MetricWire | Participant’s smartphones | 5/6 | MLM |
| Daniel et al [22] | SIAS | No | Emotion regulation  State affect | - | Participant’s smartphones | 5/6 | Other longitudinal methods |
| Daros et al [25] | SIAS | No | Single positive and negative affect ítems, emotion regulation strategies, social desire | Sensus | Participant’s smartphones | 6/14 | MLM |
| Di Matteo et al [60] | LSAS^f^ | No | Major depression disorder and generalized anxiety disorder | Android Smartphone Features | Participant’s smartphones | 14 | MLM |
| Doorley et al [28] | SIAS | No | Happiness,  anxiety, sense of belonging, social approach/avoidance | SurveySignal | Participant’s smartphones | 7/10 | MLM |
| Doorley et al [27] | SIAS | No | Emotions  Communication | SurveySignal | Participant’s smartphones | 7/10 | MLM |
| Farmer and Kashdan [61] | SIAS | No | Positive and negative emotion regularion, social events | Website link | - | 6/14 | MLM |
| Farmer and Kashdan [16] | SIAS | No | Affect and self-esteem | Online daily diary | - | 1/14 | MLM |
| Geyer et al [62] | SIAS | No | Single positive and negative affect ítems | Sensus | Participant’s smartphones | 6/14 | MLM |
| Goodman et al [63] | SIAS | 3 items of state SA | Alcohol consumption and social interaction quality | Self-applied on a secure server | - | 1/14 | MLM |
| Goodman et al [17] | ADIS, DSM-IV | No | Subjective well-being, striving | PMAT | Palm Pilot X22 | 1/14 | MLM |
| Goodman et al [18] | DSM-IV | No | Emotions, emotion regulation strategies and social situations | PMAT | Palm Pilot X22 | 1/14 | MLM |
| Goodman et al [32] | SCID | No | Anxiety intensity, controllability, regulatory strategies | - | Participant’s smartphones | 1/14 | MLM |
| Goodman et al [30] | SIAS | No | Negative affect (NA), positive affect (AP) | ESMCapture | Participant’s smartphones | 5/14 | MLM |
| Goodman et al [31] | SIAS | No | (S1) Affect, social comparisons  (S2) Affect, social comparisons, presence of other people | Self-applied on a secure server | Participant’s smartphones | (S1) 1/21  (S2) 5/14 | MLM |
| Goodman et al [29] | ADIS, DSM-IV | No | Striving  Meaning in life  Emotion suppression  Affect | PMAT | Palm Pilot X22 | 1/14 | MLM |
| Goodman et al [64] | SCID | No | Drinking motives, positive drinking consequences, drinking quantity and context | Text messages with links | Participant’s smartphones | 2/14 | MLM |
| Hannah Lee [65] | SIAS | No | State SA, judgmentalness, and unfamiliarity of interaction partner. | Web-based program | Participant’s smartphones. | 3 weeks/5 messages per day | MLM |
| Helbig-Lang et al [46] | SCID | No | PEP^j^, self-focused attention, safety behaviour use, negrative affect, maximum anxiety and situational features. | - | Blackberry device. | 7 | MLM |
| Hur et al [66] | LSAS-SR | No | Negative affect (afraid, nervous, worried, hopeless, sad), positive affect (calm, cheerful, content, enthusiastic) and social context | SurveySignal | Participant’s smartphones | 8/7 | MLM |
| Jacobson et al [47] | SIAS | No | Accelerometer  Social contact | Sensus mobile app | No | 14 | ML^h^ |
| Jacobson and Bhattacharya [67] | GAD – Q^c^ and SPDQ^p^ | Yes | Positive and negative affect, experiential avoidance, and passive sensing data. | “Mood Triggers” application | Participant’s smartphones | 1hr/7 | Deep Learning Model |
| Kane and Ashbaugh [68] | SPIN | Yes | No | OhDontForget.com and Qualtrics | Participant’s smartphones | 3/2 | MLM |
| Kashdan and Steger [69] | SCID - ADIS | 7 items | Daily: emotional regulation, positive and negative affect and positive activities | PMAT | Palm Pilot Z22 | 14 | MLM |
| Kashdan and Collins [70] | SCS^n^ | No | Emotions, social context and  activity | - | PSION Organizer II LZ 64 | 4/14 | MLM |
| Kashdan et al [71] | SIAS | No | Pleasure  Closeness and conectness  Sex activity | Online website | - | 21 | MLM |
| Kashdan et al [20] | SCID and ADIS | No | Emotions and social interaction | PMAT | Palm Pilot Z22 | 14 | MLM |
| Kashdan et al [21] | DSM-IV | 3 items | Experiential avoidance | PMAT | Palm Pilot Z22 | 14 | MLM |
| Kashdan and Farmer [19] | SCID and SIAS | No | Momentary emotion, social interaction  And end-of-day emotion | Online website | - | 5/14 | MLM |
| Katz et al [41] | SCID, SIAS and SPS | No | PEP | Self-applied | Personal Digital Assistant (PDA) | 4/14 | MLM |
| Kivity and Huppert [72] | MINI, SIAS and SPS | No | Alcohol use, suppression, rumination and avoidance | Unclear | - | 4/7 | MLM |
| Kim and Kwon [73] | MINI and SAD | SPIN | Average and peak of daily SA. | Text message reminder | - | 1/7 | MLM |
| Ladis et al [26] | SIAS | No | Positive and negative affect, emotion regulation strategies, motivation to change emotions, and subjective effectiveness | Sensus | Participant’s smartphones | 14 weeks/6 per day | MLM |
| Lee et al [42] | SPAI^s^ | An index of SA was calculated by seven emotion ratings. | - | - | - | 3/10 | ANOVA |
| Nanamori et al [74] | LSAS | No | Perception of stimuli in a social situation, fear of stimuli, self-focused attention from the observer perspective | Not specified | Participant’s smartphones | 10 days | MLM |
| Naragon-Gainey [43] | ADIS | IDAS | Positive affect and negative affect | SurveySignal | Participant’s smartphones | 3/10 | MLM |
| O’Grady et al [75] | Self-Consciousness Scale | Feeling awkward or embarrassed in public | Alcohol use | Self-applied - website | - | 1/30 | MLM |
| Oren-Yagoda et al [35] | ADIS and LSAS | No | Momentary emotion experiences  Momentary social events | Daily Events Survey | Participant’s smartphones | 21 | MLM |
| Oren-Yagoda and Aderka [33] | ADIS and LSAS | No | Momentary emotion experiences  Momentary social events | Not specified | Participant’s smartphones | 21 | MLM |
| Oren-Yagoda et al [36] | ADIS and LSAS | No | Momentary emotion experiences  Momentary social events | Not specified | Participant’s smartphones | 21 | MLM |
| Oren-Yagoda et al [34] | ADIS and LSAS | No | Momentary emotion experiences  Momentary social events | Not specified | Participant’s smartphones | 21 | MLM |
| O’Toole et al [37] | LSAS | 7 items of SA | Emotion differentiation negative and positive affect. Satisfaction with social life | E-mail link to the survey | Personal computer | 11 | MLM |
| O’Toole et al [38] | LSAS | 7 items of SA | Positive and negative affect. Emotion regulation strategies | E-mail link to the survey | Personal computer | 11 | MLM |
| Papp et al [76] | IDAS | No | Negative and positive mood | App designed specifically for the research | iPod Touch | 28/5 | MLM |
| Park and Naragon-Gainey [45] | ADIS | IDAS | Emotional clarity  ER success | SurveySignal | - | 10 | MLM and SEM |
| Piccirillo and Robedaugh [77] | MINI-5 | No | Major depressive disorder | Not specified | Participant’s smartphones | 5/30 | Multilevel and person-specific vector autoregression (VAR) |
| Reichenberger et al [78] | No | - | S1: Stressor type, positive and negative affect  S2: Stressor type, negative and positive emotions | PsyDiary | Participant’s smartphones | 5/6 | MLM |
| Rinner et al [40] | SCID | Single ítem from Kashdan & Steger (2006) | Sadness, happiness, physical activity | - | Smartphone provided by researchers | 5/7 | MLM |
| Russell et al [44] | MINI, LSAS and SIAS | No | Interpersonal behavior, emotions during interaction | Unclear | Unclear | 14 calls in 5 days | MLM |
| Saulnier et al [79] | SCID | No | Anxious arousal, anxious apprehension | Not specified | Participant’s smartphones or email. | 2 weeks, 5 times per day | DSEM^b^ |
| Seah et al [48] | S1: ADIS  S2: LSAS | No | Social avoidance and experience of positive and negative emotions | S1: Purdue Momentary Assessment Tool  S2: Qualtrics | S1: Palm Centro  S2: Participant’s smartphones and/or computers. | S1: 5/14  S2: 2/10 | MLM |
| Villanueva et al [39] | SCID | No | Social interaction  Positive affect and negative affect | - | Smartphone provided by researchers | 6/7 | MLM |
| Walukevich-Dienst et al [80] | SIAS | No | Alcohol and cannabis use-related consequences. | Not specified | Not specified | 14 days/1 per day | MLM |
| Wilson et al [81] | MINI and SPIN | No | Positive and negative feedback seeking. | E-mails | - | 14 | Unclear |

^a^ADIS: Anxiety Disorder Interview Schedule

^b^DSEM: Dynamic Structural Equation Modeling

^c^GAD-Q: Generalized Anxiety Disorder Questionnaire

^d^IDAS: Inventory of Depression and Anxiety Symptoms

^e^LMM: Linear Mixed Models

^f^LSAS: Liebowitz Social Anxiety Scale

^g^MINI: Mini International Neuropsychiatric Interview

^h^ML: machine learning

^i^MLM: multilevel modeling

^j^PEP: post-event processing

^k^PMAT: purdue momentary Assessment Tool

^l^SCID-I/NP: Structured Clinical Interview for DSM-IV Axis I Disorders

^m^SCID: Structured Clinical Interview for DSM-IV

^n^SCS: Self-Consciousness Scale.

^o^SIAS: Social Interaction Anxiety Scale

^p^SPDQ: Social Phobia Diagnostic Questionnaire

^q^SPIN: Social Phobia Inventory.

^r^SPS: Social Phobia Scale

^s^SPAI: Social Phobia and Anxiety Inventory
